# Megaripple Migration on Mars

**DOI:** 10.1029/2020JE006446

**Published:** 2020-07-29

**Authors:** S. Silvestro, M. Chojnacki, D. A. Vaz, M. Cardinale, H. Yizhaq, F. Esposito

**Affiliations:** ^1^ INAF Osservatorio Astronomico di Capodimonte Napoli Italy; ^2^ SETI Institute Mountain View CA USA; ^3^ Lunar and Planetary Laboratory University of Arizona Tucson AZ USA; ^4^ Planetary Science Institute Tucson AZ USA; ^5^ Centre for Earth and Space Research of the University of Coimbra Observatório Geofísico e Astronómico da Universidade de Coimbra Coimbra Portugal; ^6^ DiSPUTer D'Annunzio University Chieti Italy; ^7^ Department of Solar Energy and Environmental Physics, BIDR Ben‐Gurion University of the Negev Beersheba Israel

**Keywords:** Ripples, Mars, Aeolian, Dune, Migration, Megaripples

## Abstract

Aeolian megaripples, with 5‐ to 50‐m spacing, are abundant on the surface of Mars. These features were repeatedly targeted by high‐resolution orbital images, but they have never been observed to move. Thus, aeolian megaripples (especially the bright‐toned ones often referred as Transverse Aeolian Ridges—TARs) have been interpreted as relict features of a past climate. In this report, we show evidence for the migration of bright‐toned megaripples spaced 1 to 35 m (5 m on average) in two equatorial areas on Mars indicating that megaripples and small TARs can be active today. The moving megaripples display sand fluxes that are 2 orders of magnitudes lower than the surrounding dunes on average and, unlike similar bedforms on Earth, can migrate obliquely and longitudinally. In addition, the active megaripples in the two study areas of Syrtis Major and Mawrth Vallis show very similar flux distributions, echoing the similarities between dune crest fluxes in the two study areas and suggesting the existence of a relationship between dune and megaripple fluxes that can be explored elsewhere. Active megaripples, together with high‐sand flux dunes, represent a key indicator of strong winds at the surface of Mars. A past climate with a denser atmosphere is not necessary to explain their accumulation and migration.

## Key Points

- Bright‐toned megaripple migration is identified on the surface of Mars for the first time
- These features are similar to Transverse Aeolian Ridges, which had been assumed to be dormant under the current climate
- Megaripple crest fluxes are, on average, 2 orders of magnitude lower than local dune crest fluxes

## 1 Introduction and Study Areas

**Figure 1:**
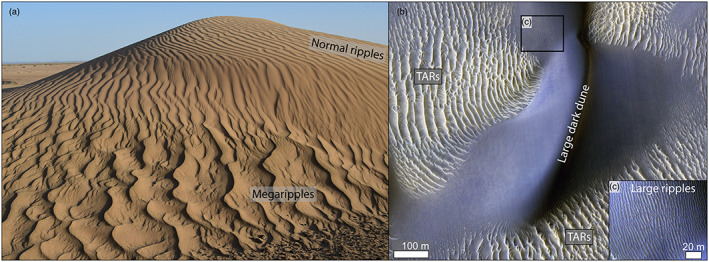
Aeolian bedforms on Earth and Mars. (a) Normal ripples and megaripples over a dune in Morocco (Merzouga). Normal ripples are ~20–25 cm spaced, while megaripples are ~60–70 cm spaced. Photo by H. Yizhaq. (b) Aeolian bedforms in Proctor crater on Mars where large ripples (LRs) sculpt the slope of a dune. Bright TARs are visible in the interdune. HiRISE PSP_003800_1325, NASA/ JPL/University of Arizona.

Aeolian sand ripples are widely studied to constrain the wind environment at the surface of Mars and to interpret its stratigraphic record (Banks et al., 2018; Diniega et al., 2017; Lapotre et al., 2016a; Vaz & Silvestro, 2014). Due to the peculiar physical characteristics of the Martian atmosphere (Kok et al., 2012; Lapotre et al., 2018; Siminovich et al., 2019; Sullivan et al., 2018), ripples on Mars are 1 order of magnitude larger than their terrestrial counterparts and are thus visible on high‐resolution submeter orbiter images (Bridges et al., 2007). Thus, the aeolian environment in many areas of Mars can be characterized remotely and key geological parameters such as sand flux and erosion rates can be computed (Banks et al., 2018; Bridges et al., 2012a, 2017; Chojnacki et al., 2019; Diniega et al., 2017; Runyon, Bridges, & Newman, 2017). On the other hand, with sparse in situ sedimentological data, the characterization and classification of the bedforms covering the surface of Mars is much more challenging. For instance, a proper granulometrical analysis is key to distinguish two classes of ripples on Earth: normal ripples and megaripples (Figure 1) (Greeley & Iversen, 1985; Lancaster, 1995; Yizhaq, Katra, Isenberg, & Tsoar, 2012; Yizhaq et al., 2019). Normal terrestrial ripples, up to 30 cm spaced, form in unimodal sand (0.1–0.3 mm), while megaripples, 30 cm to 43 m spaced, form in in bimodal sand and are located where the coarser sand fraction tends to accumulate between dunes (Figure 1) (Bagnold, 1954; Milana, 2009; Sharp, 1963; Yizhaq, Katra, Kok & Isenberg, 2012; Yizhaq et al., 2019). Coarse grains (>1 mm) accumulating on the crest of megaripples form a stable wind‐resistant armoring layer so, despite their smaller size compared to larger bedforms such as sand dunes, megaripples are less mobile (Hugenholtz & Barchyn, 2017; Isenberg et al., 2011; Yizhaq & Katra, 2015). Megaripple dynamics have only been monitored at a few sites on Earth and their migration can be associated with very strong wind events (Hugenholtz & Barchyn, 2017; Isenberg et al., 2011; Lorenz & Valdez, 2011; Milana, 2009; Sakamoto‐Arnold, 1981; Yizhaq et al., 2019; Zimbelman et al., 2009). On Mars, aeolian bedforms of large ripples (LRs) and Transverse Aeolian Ridges (TARs) are typically classified based on measurements of wavelength and albedo through remote sensing, as sedimentological data from the surface are limited (Bourke et al., 2010; Lapotre et al., 2016a; Malin & Edgett, 2001; Silvestro et al., 2016a; Zimbelman et al., 2013).

Martian LRs are 1–5 m spaced and 0.1–0.4 m tall (Lapotre et al., 2018; Silvestro et al., 2016a; Sullivan et al., 2008). LRs normally sculpt the slopes of sand dunes or accumulate as isolated patches in topographic lows (Bridges et al., 2007; Sullivan et al., 2008). LRs, active in the present‐day Martian climatic setting (Bridges et al., 2013; Geissler et al., 2012; Silvestro et al., 2010), have been interpreted as normal impact ripples (Sullivan et al., 2008; Sullivan & Kok, 2017) or fluid‐drag ripples (Lapotre et al., 2016a, 2018). Bright‐toned bedforms with wavelength ranging from ~6 to ~140 m and height ~0.3 to ~6.4 m were initially named TARs as the exact process behind their formation has been debated (Bourke et al., 2003; Geissler, 2014; Geissler & Wilgus, 2017; Hugenholtz et al., 2017; Shockey & Zimbelman, 2012; Zimbelman et al., 2013). When found together, dark dunes overlay and often migrate over TARs and, despite inspection for activity within image pairs spaced up to 2 or 3 MYs by numerous researchers, they have never been reported to migrate (Balme et al., 2008; Banks et al., 2018; Berman et al., 2018; Bridges, Bourke, et al., 2012; Bridges et al., 2013; Chojnacki et al., 2014). Thus, TARs have been widely considered to be remnant landforms from a prior climate and obliquity, a scenario confirmed in several areas of Mars by the local superposition of craters and fractures (Berman et al., 2018; Edgett & Malin, 2000; Kerber & Head, 2012; Malin & Edgett, 2001; Reiss et al., 2004; Sullivan et al., 2008; Zimbelman et al., 2013). Certain smaller bedforms with wavelengths of 5–15 m overlap with small TARs and have been commonly interpreted as megaripples. These bedforms have lower albedo than TARs and are often found together and in continuity with dark dunes (Fenton, 2005; Silvestro et al., 2011; Zimbelman, 2010, 2019). Thus, megaripples are considered younger than larger TARs, where saltating sand is suspected to keep them active in the long term (Balme et al., 2008; Berman et al., 2011; Silvestro et al., 2011; Zimbelman, 2019). It may be the presence of moving dunes, providing a source of saltators capable of displacing larger grains by impact‐driven creep (Kok et al., 2012), seems a necessary condition to keep the coarser grained megaripples active (Berman et al., 2011). However, at the time of this writing, only limited orbital evidence for Martian megaripple activity has been reported (Chojnacki et al., 2019); thus the degree, mode, and context of Martian megaripple dynamics are unknown at present (Berman et al., 2011, 2018; Chojnacki et al., 2019). It should be emphasized that a clear distinction between megaripples and TARs is frequently absent as these features can be found in continuity often with a wide range of albedo (Silvestro et al., 2011; Zimbelman, 2010, 2019).

**Figure 2:**
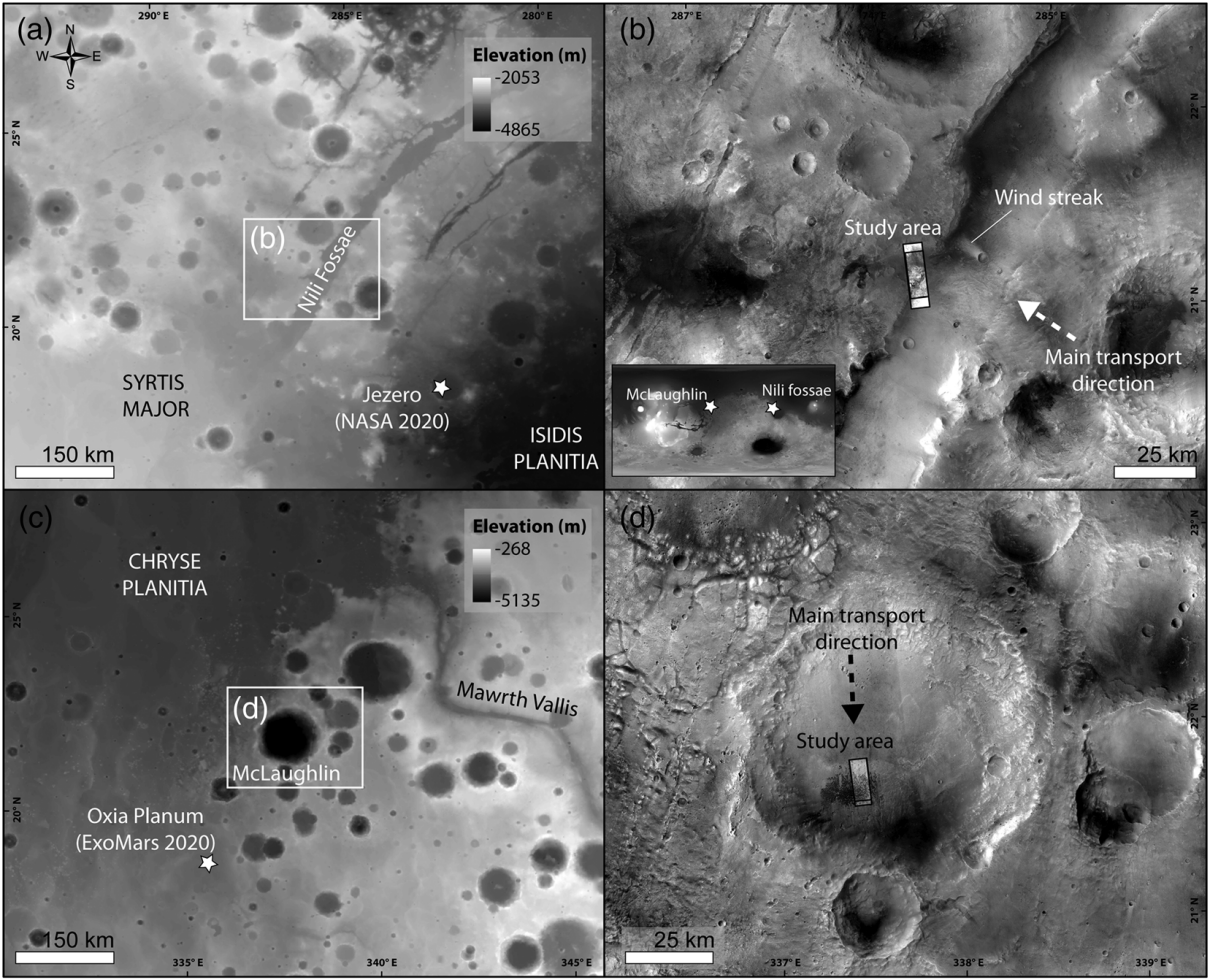
Study area. (a) Nili Fossae regional context. (b) Nili Fossae HiRISE image footprints location. (c) McLaughlin regional context. (d) McLaughlin HiRISE image footprints location. (a, c) MOLA elevation gridded map and (b, d) CTX image mosaic.

**Figure 3:**
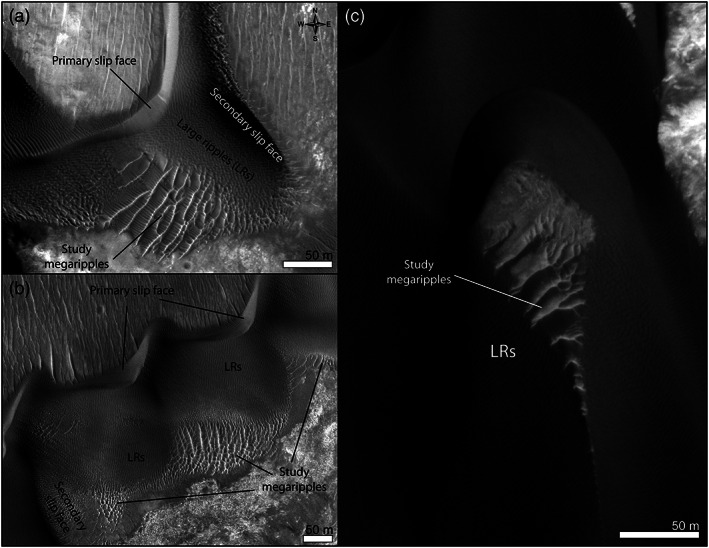
Study bedforms. (a, b) Nili Fossae. (c) McLaughlin crater. (a, b) HiRISE ESP_047094_2015 and (c) PSP_009814_2020.

**Figure 4:**
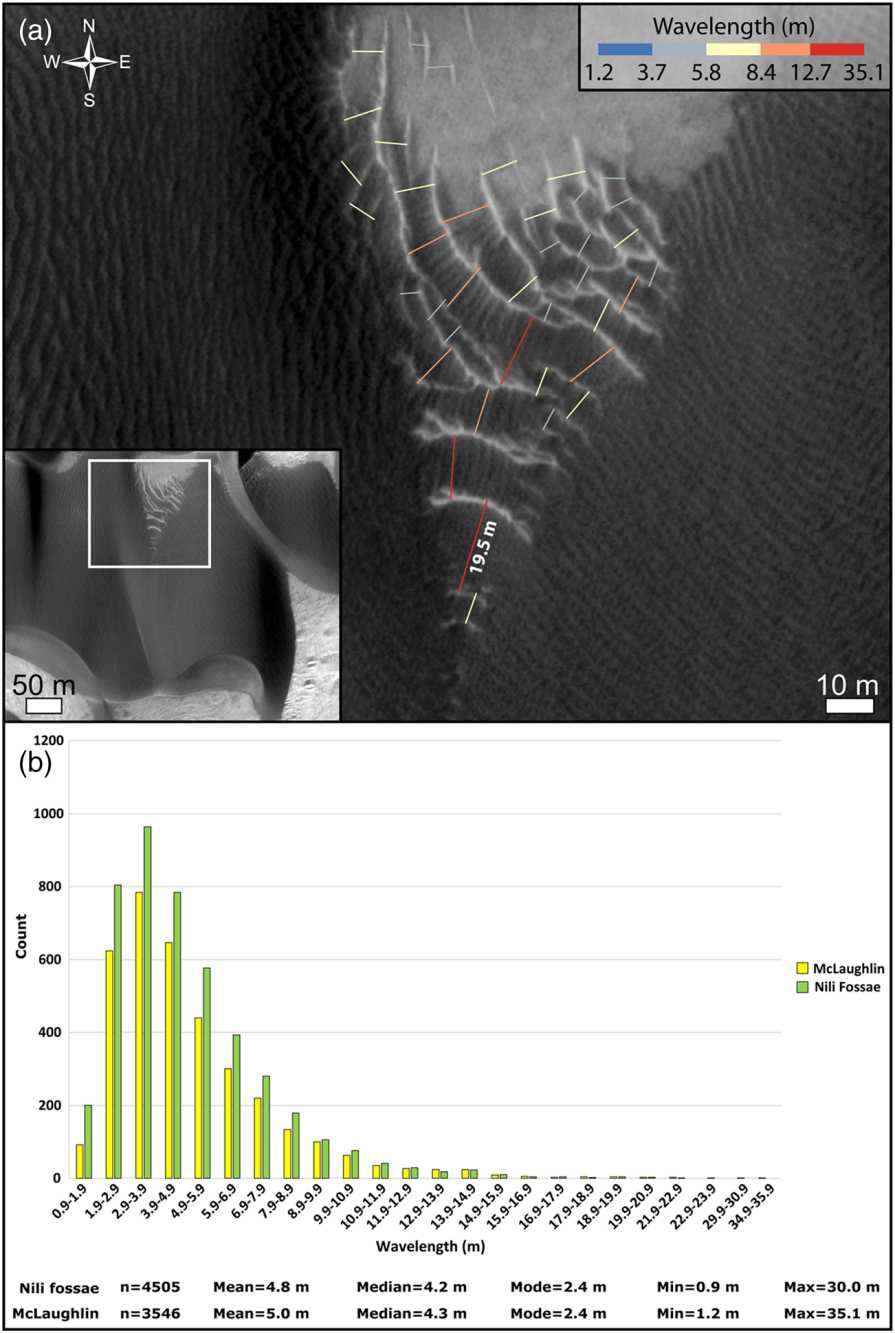
Megaripple wavelength in the study areas. (a) Example of wavelength manual computation over a field of megaripples in McLaughlin crater. (b) Wavelength distributions for Nili Fossae and McLaughlin megaripples. HiRISE ESP_045312_2020.

Here we report on dune‐associated sand ripples located in Nili Fossae and McLaughlin crater, which show bedform spacings of up to 35 m (5 m on average) and a range of albedos (moderate to bright toned) (Figures 2, 3, 4). Based on the study bedforms' greater spacing and perceived greater albedo relative to decameter‐scale dark ripples, along with comparisons with terrestrial megaripples that are also found in stoss or interdune areas (Sharp, 1963), we will refer to these as megaripples hereafter (Hugenholtz et al., 2017; Zimbelman, 2010). The study megaripples are located close to high‐flux dunes in Nili Fossae (∼200 km NW from the National Aeronautics and Space Administration (NASA) 2020 Rover landing site, Figures 1a and 1b) and McLaughlin crater (∼200 SW from the ESA‐ROSCOSMOS ExoMars Rover landing site, Figures 2c and 2d) (Bhardwaj et al., 2019; Chojnacki et al., 2018, 2019; Pajola et al., 2017). Dunes in Nili Fossae are migrating toward the NW wall of an ~600‐m‐deep graben and are interpreted to be driven by anabatic northeasterly winds blowing from the Isidis Basin (Figures 2a and 2b) (Chojnacki et al., 2018, 2019). McLaughlin crater is an ~2‐km‐deep Noachian impact basin situated at the dichotomy boundary SE of Chryse Planitia (Figure 2c). Dunes and megaripples are accumulated in the southern portion of the crater floor by dominant winds from the N in an area of increased surface roughness due to the ejecta deposit of an ~25‐km crater nearby (Figure 2d) (Davis et al., 2019). By analyzing a series of multitemporal High Resolution Imaging Science Experiment (HiRISE) images (McEwen et al., 2007) (Table 1), we test the hypothesis that megaripples associated with active dunes could be active in the present‐day atmospheric setting.

**Table 1:** Image Acquisition Parameters for the HiRISE Images Used to Track Dune and Megaripple Migrations

*Nili fossae (ΔT = 9.38 Earth years)*		
Orbit ID	PSP_003086_2015_RED_A_01_ORTHO (T1)	ESP_047049_2015_RED_A_01_ORTHO (T2)
Source image ID	PSP_003086_2015	ESP_047049_2015
Acquisition Date	24 March 2007	9 August 2016
Local Mars Time	15:38	15:21
Resolution	25 cm/pixel	25 cm/pixel
Emission angle	7.4°	0.3°
Phase angle	55.5°	57.6°
Solar incidence angle	62°	58°
Solar longitude	206.4°, northern autumn	200.8°, northern autumn
Subsolar azimuth	339.6°	339.7°
*McLaughlin Crater (ΔT = 7.57 Earth years)*		
Orbit ID	PSP_009814_2020_RED_A_01_ORTHO (T1)	ESP_045312_2020_RED_A_01_ORTHO (T2)
Source image ID	PSP_009814_2020	ESP_045312_2020
Acquisition date	30 August 2008	27 March 2016
Local Mars Time	15:26	15:07
Resolution	25 cm/pixel	25 cm/pixel
Emission angle	1.6°	0.9°
Phase angle	49.2°	42.8°
Solar incidence angle	48°	44°
Solar longitude	119.9°, northern summer	128.4°, northern summer
Subsolar azimuth	16.3°	11.0°

## 2 Materials and Methods

**Figure 5:**
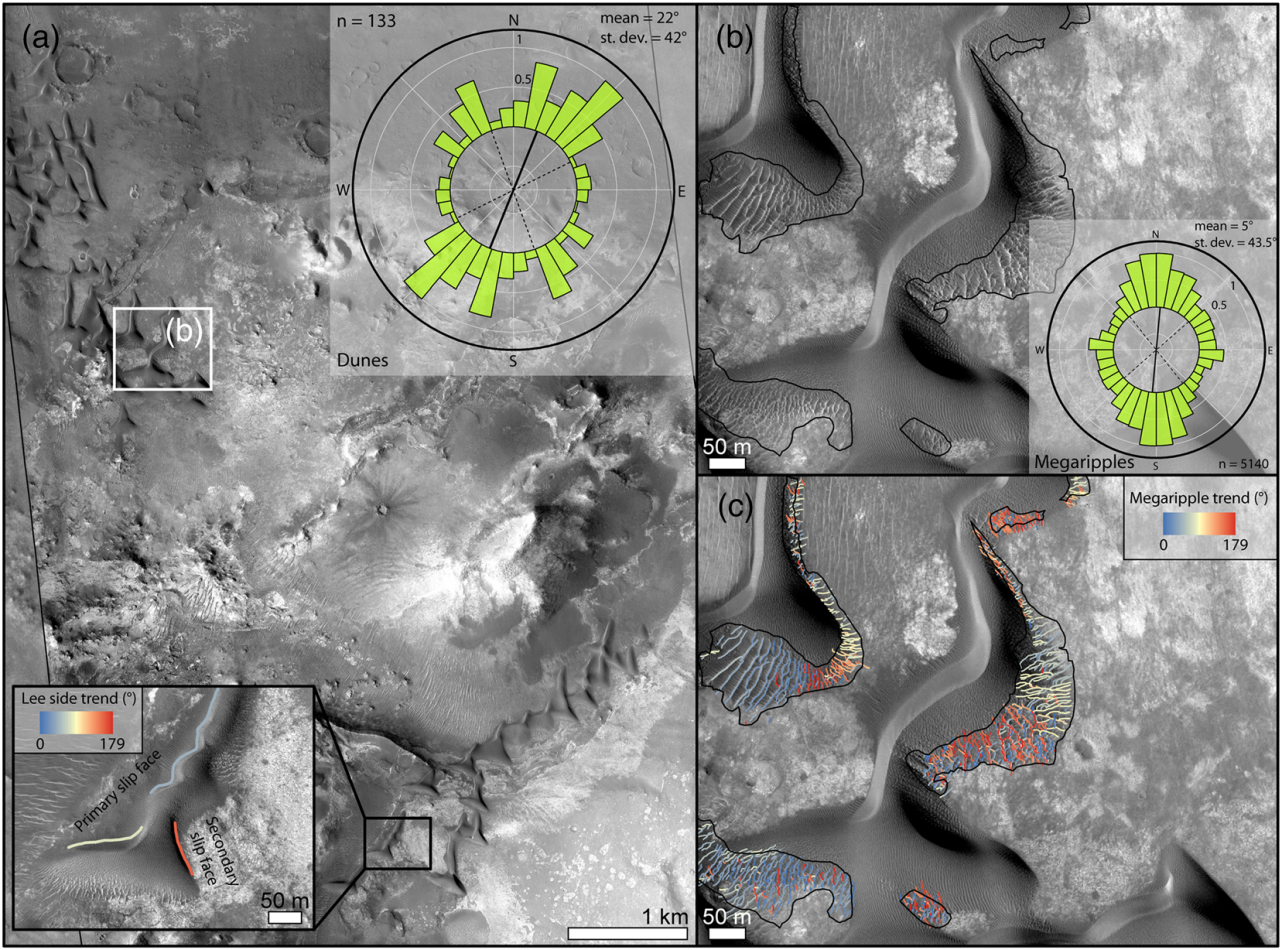
Bedform morphology in Nili Fossae. (a) Dunes show two slip faces with different trends (inset on the bottom left) showing a complex arrangement (see circular plot on inset at the top right). (b) Megaripple areas mapped on HiRISE and distribution of the crestline trends (inset). (c) Mapped megaripples with the crests colorized by their trend values. (a–c) HiRISE ESP_047094_2015.

Dune and megaripple morphologies were studied on HiRISE images (0.25–0.5 m/pixel) (McEwen et al., 2007) (Table 1). Background images were Context Camera (CTX) image mosaics (Malin et al., 2007) downloaded at http://murray-lab.caltech.edu/CTX/tiles/beta01/ (Dickinson et al., 2018). Ripple wavelengths (***w***_***r***_) were computed by manually tracing the perpendicular line connecting subsequent ripple crestlines in ArcMap® (Figure 4). Ripple half‐height (***h***_***r***_) was then derived using the relationship ***h***_***r***_ ***= w***_***r***_/**20** (Bridges et al., 2012b; Runyon, Bridges, & Newman, 2017). Dune lee faces were mapped as lines at the base of the dunes and megaripple crestlines were mapped in ArcMap® on the Time 1 (T1) images (Table 1) in both study areas and the directional trend (0–179°) derived for each of the mapped lines (Figure 5). Megaripple and dune trends were plotted at the edge of a circular diagram using the programming language R. In the center of the diagram, we plotted statistical parameters for megaripple morphology, such as circular directional mean and standard deviation (Figures 5a and 5b insets).

Dune migration was computed semiautomatically in a Geographic Information System (GIS) environment using overlapping HiRISE images of the study areas. The HiRISE image pairs were acquired with similar season and lighting parameters making them suitable for change detection (Table 1).

**Figure 6:**
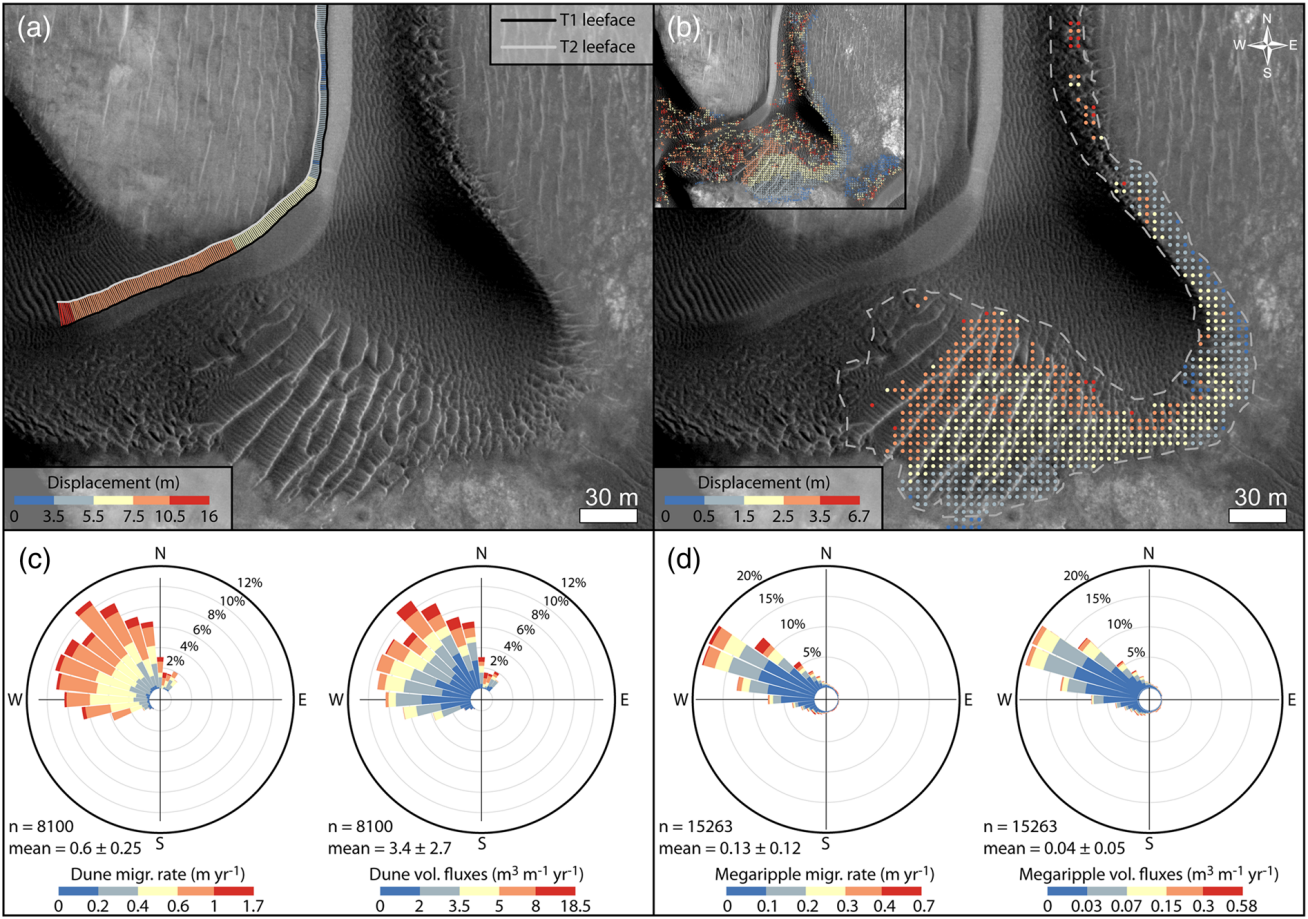
Bedform migration in Nili Fossae (see also Animation S2). (a) Example of dune displacement computation. (b) Example of COSI Corr megaripple displacement measurement (uncleaned data in inset). (c) Circular plot showing the directional and magnitude distribution of the dune migration vectors (rates and fluxes) shown in panel (a). (d) Circular plot showing the directional and magnitude distribution of the megaripple rate and flux vectors. (a, b) HiRISE ESP_047094_2015.

In Nili Fossae images were used from Earth year (EY) 2007 (T1, Martian year—MY 28) and 2016 (T2, MY 33) (Δ*T* = 3,425 days = 9.38 EY) while in McLaughlin crater from 2008 (T1, MY 29) and 2016 (T2, MY 33) (Δ*T* = 2,765 days = 7.57 EYs) (Table 1). The bases of dune lee sides over the T1 and T2 HiRISEs were mapped and the displacement vectors connecting the mapped lines derived assuming a perpendicular migration (Figure 6a) (Cardinale et al., 2016; Vaz et al., 2015; Vaz & Silvestro, 2014). Migration vectors were then converted to flux vectors by multiplying the migration rates to the dune heights derived from HiRISE digital terrain models (DTMs) at the slip face brink heights following the method of Urso et al. (2018) leading to the dune crest fluxes in m^3^ m^−1^ yr^−1^ as defined by Vermeesch and Drake (2008). Note that this method of computing total sand fluxes, avoiding to track small slipfaceless dunes (Davis et al., 2019), is different than other authors and is not directly comparable (Bridges et al., 2012a; Chojnacki et al., 2018, 2019; Runyon, Bridges, & Newman, 2017). The process of orthorectification of the monitoring images was performed in SOCET SET® BAE Systems photogrammetry software (Kirk et al., 2008) following well established methods and procedures (Chojnacki et al., 2018). HiRISE DTMs and image are summarized in Table S1 in the supporting information.

Dune, megaripple, and ripple changes were manually inspected using overlapping HiRISE long‐baseline (5 MYs) orthoimages. Ripple migration was quantified by using the “Co‐registration of Optically Sensed Images and Correlation” (COSI‐Corr) tool suite (Leprince et al., 2007), which produces a dense vectorial map of ripple migration (Ayoub et al., 2014; Bridges et al., 2012a; Cardinale et al., 2016). Ripple displacement maps at the resolution of 4 m/pixel were produced by using the T1‐T2 image pairs (Table 1) using a window size of 70 pixels, a step of 16 pixels, and a search range of 15 pixels, ideal values for detecting ripple displacements on HiRISE data (Figure 6b) (Bridges et al., 2012a; Cardinale et al., 2016; Silvestro et al., 2016a). The COSI‐Corr maps were further improved by removing long‐wavelength jitter artifacts by computing jitter residuals on bedrock areas and by subtracting these values to the masked dune area (Figure S1) (Silvestro et al., 2016a, 2016b). However, due to the long time span covered by the HiRISE data, and the high fluxes of the selected sites (Chojnacki et al., 2018, 2019), the smaller LRs migrated too fast to be tracked by COSI‐Corr. Thus, LR displacement measurements over the dune slopes are noisy (or absent) (Figure 6b inset). We filtered out most of these bad data using the signal‐to‐noise ratio (SNR) map, which is an output of the COSI‐Corr correlation process and by manually selecting suitable measurements, which, in our case, are associated to the relatively slow migrating megaripples (Figure 6b). Using this method, we were able to measure megaripple migrations at the subpixel scale (<0.25 m) (Ayoub et al., 2014; Bridges et al., 2012a). COSI‐Corr displacement data were then used to derive megaripple migration rates (m yr^−1^), whereas fluxes (m^3^ m^−1^ yr^−1^) were obtained by multiplying the migration rates to the previously mentioned average heights (Table 2). Megaripple patches were mapped in discrete polygons within ArcMap (Figure S2a) and displacement magnitudes derived with COSI‐Corr. These results in turn were further compared with manual measurements computed by tracing the T1‐T2 megaripple crestlines on 20% of the megaripple patches for a total of 1,413 polygons (344 in Nili Fossae and 1,069 in McLaughlin) (Table 2 and Figure S2). Dune and megaripple displacement vectors were then plotted on rose diagrams using the prebuilt package Openair in R (Figures 6c and 6d).

**Table 2:** Important Statistical Parameters for Displacements, Migration Rates and Fluxes for Dunes and Megaripples in the Two Study Areas

			Displacement (m)				Migration rate (m yr^−1^)				Flux (m^3^ m^−1^ yr^−1^)			
		*N*	Mean	Median	*σ*	DispUnc.^a^	Mean	Median	*σ*	RateUnc.^b^	Mean	Median	*σ*	FluxUnc.^c^
Dunes	Nili Fossae	8,100	5.57	5.31	2.35	0.50	0.59	0.57	0.25	0.05	3.42	2.71	2.74	0.44
	McLaughlin	74,241	2.62	2.37	1.40	0.50	0.35	0.31	0.19	0.07	3.39	2.97	2.24	0.76
Megaripples	Nili Fossae (cosi)	15,263	1.25	0.82	1.14	0.25	0.13	0.09	0.12	0.027	0.04	0.02	0.05	0.01
	Nili Fossae (manual)	11,063	1.81	1.50	1.45	0.25	0.19	0.16	0.15	0.027	0.07	0.04	0.08	0.01
	McLaughlin (cosi)	17,275	0.90	0.56	0.90	0.25	0.12	0.07	0.12	0.033	0.04	0.02	0.05	0.01
	McLaughlin (manual)	23,709	1.15	0.93	0.96	0.25	0.15	0.12	0.13	0.033	0.05	0.03	0.07	0.01

*Note*. Statistical values on this table are computed from the whole vectorial data set for dunes and megaripples. This is the reason of the small discrepancy on dune median flux data with the plot of Figure 9 where only averages for each dune lee foot are considered.

^a^ An uncertainty of two pixel is assumed when mapping dune lee foots on HiRISE, while one pixel is assumed when mapping megaripple crests. Lee foot mapping is considered more complicated due to grainfall structures that can bias the mapping.

^b^ Computed as DispUnc/Δ*T*.

^c^ Flux uncertainties are computed by error propagation according to $\left|\text{Flux}\right|\times \sqrt{\left({\left(\frac{\text{RateUnc}}{\text{MigrRate}}\right)}^{2}+{\left(\frac{\text{Zunc}}{Z}\right)}^{2}\right)}$ where *Z* is the bedform height and Z_unc_ is the assumed height uncertainty (0.50 m). Note that the flux uncertainty integrates the manual mapping errors (mapping the slip face exactly on its edge) and the vertical precision of the HiRISE DTMs (tens of centimeters as stated in the HiRISE website, https://www.uahirise.org/dtm/about.php).

## 3 Results

### 3.1 Nili Fossae Bedforms

Dunes in Nili Fossae have a barchanoid form with two slip faces, which yield a triangular shape to the dunes (Figures 3a, 3b, and 5a inset). Dunes are 5.5 m tall on average (measured on HiRISE DTM) and up to 260 m in length. The main dune slip faces dip toward the NW and together with the orientation of the wind streaks indicate the primary formative winds are blowing from the SE (Figures 2b, 3a, and 3b). However, the presence of additional slip faces trending NE–SW (inset in Figure 5a) indicates a more complex wind regime. On average, dune trends at 22 ± 42° (all reported uncertainties correspond to 1*σ*). The high dispersion of the data is caused by the secondary slip face trends. Megaripples in Nili Fossae are located along the flanks and stoss slopes of the dark dunes and are 1–117 m in cross‐crest length (12.6 ± 10.4 m) (Figures 5b and 5c). In general, megaripple crests are slightly brighter in albedo than nearby dunes and their crests continuously merge with LR crests (Figures 3a and 3b). Megaripple trends are multimodal (5 ± 43.5°) as they appear to be strongly influenced by the dune topography (Figures 5b, 5b inset, and 5c). In term of albedo and topographic setting, megaripples in Nili Fossae can be considered analogs to the megaripples described in Meridiani Planum, Gamboa and Matara craters (Silvestro et al., 2011; Zimbelman, 2019).

In the T1‐T2 HiRISE image pair (Table 1), the migration of dunes, megaripples, and LRs are evident (Figure S3 and Animations S1–S4). During the ~9.4 EYs spanned by the T1 and T2 HiRISE, dunes displaced on average 5.6 m toward the NW (0.2 to 16 m) (Figure 6a), while megaripples 1.2 m on average (6.7 m maximum) (Figure 6b). These values translate to average migration rates of 0.6 and 0.13 m yr^−1^ for dunes and megaripples, respectively (Figures 6c and 6d and Table 2). On average megaripples in Nili Fossae are moving more than 4 times slower than dunes, despite the former's significantly smaller height and volume. The megaripple fluxes (0.04 m^3^ m^−1^ yr^−1^) are on average 2 orders of magnitude lower than the dune crest flux (3.4 m^3^ m^−1^ yr^−1^). This is the first time that megaripple movement is documented on Mars and that dune and megaripple migration rates and fluxes are compared in the same area. Nili Fossae dune and megaripple displacement vectors are compatible where both are migrating toward the WNW (Animations S1 and S2). Some megaripples do not display a pure transverse migration as they are migrating obliquely or longitudinally (Animations S3 and S4). Other bedforms, lying below the dune sand, are completely static (Animations S1, S2, and S4).

### 3.2 McLaughlin Crater Bedforms

**Figure 7:**
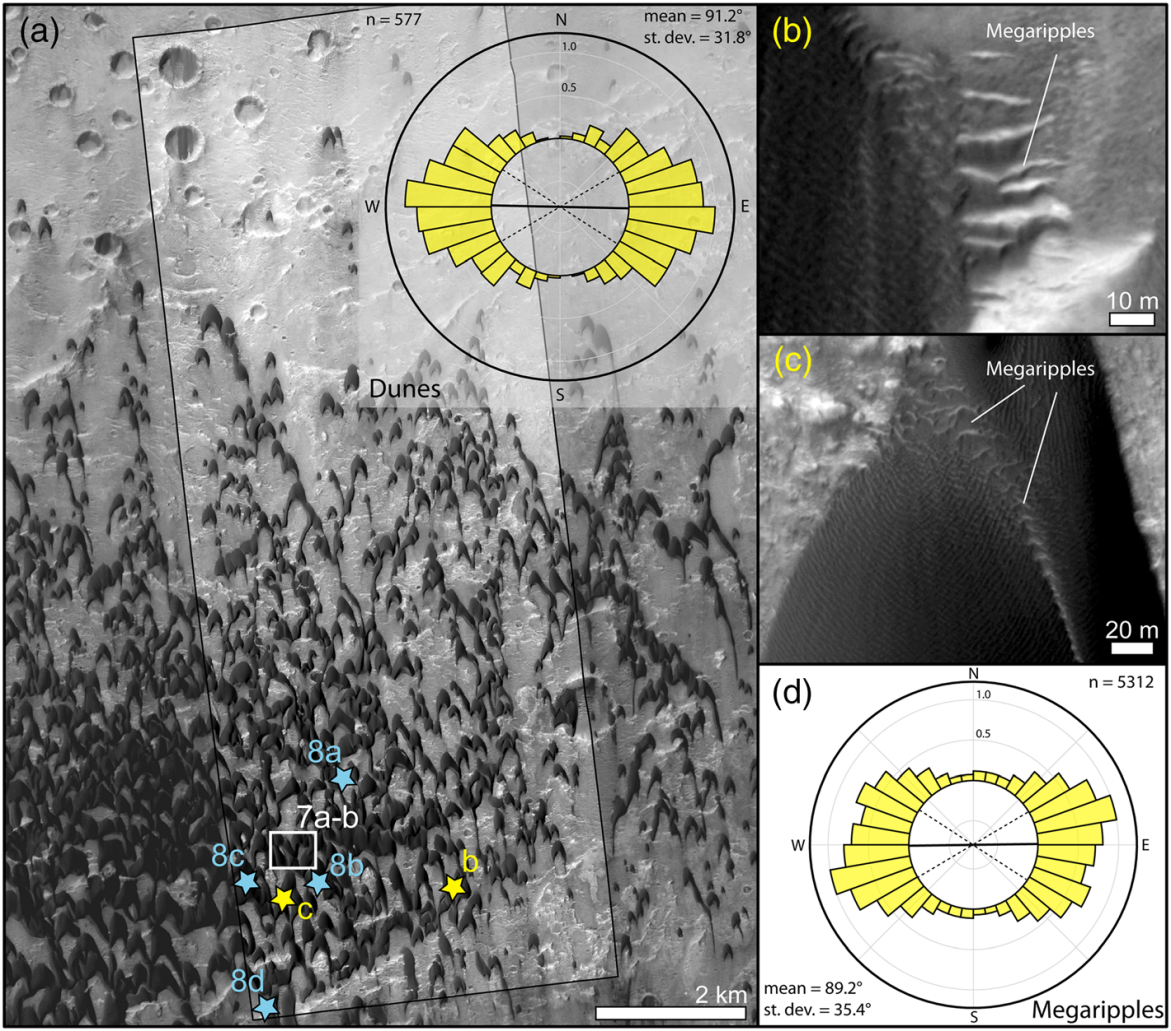
Bedform morphology in McLaughlin crater. (a) Study area and directional distribution of the mapped lee sides (inset). (b) Bright‐toned active megaripples partially covered by the dune sand. (c) Active megaripples lagging behind the stoss side of a dune, unlike the panel b examples, these megaripples are part of the main dune body. (a–c) HiRISE ESP_045312_2020.

Dunes in McLaughlin crater are asymmetric barchan and barchanoid in morphology with slip faces' trending EW at 91.2 ± 31.8° (Figures 7a, 7a inset, and S4 and Animation S5). Such a crestline arrangement indicates dune‐shaping winds are mainly blowing from the north. Dunes are 11 m tall on average and up to 300 m in length (Davis et al., 2019). Colocated with the dunes, megaripples with variable albedo are visible upwind and in between dunes (Figures 3c, 7b, and 7c). Megaripples are 0.5–100 m in length (8 ± 6.5 m) and can be much brighter than dune sand (Figure 3c). Many active bright‐toned megaripples are located stratigraphically below the dunes and their crests do not merge with the neighboring LRs (Figures 3c and 7b). Similar bedforms have been classified in other works as TARs (Zimbelman, 2010, 2019). Other megaripples in McLaughlin are darker and are part of the main dune body, with their crestlines continuously merging with nearby LRs (Figure 7c). These latter examples are similar to the ones described in Nili Fossae and in other areas of Mars (Silvestro et al., 2011; Zimbelman, 2010, 2019). However, the scenario described here is an oversimplification. A clear distinction between bright‐ and dark‐toned megaripples is not always possible due to the infilling of the dark sand in between megaripple crests, which also complicates the interpretation of the stratigraphic relationships between dunes and megaripples. In addition, more complicated bedform arrangements occur that deviate from what appears to be typical (e.g., bright‐toned megaripples in continuity with dune sand, Animation S6).

**Figure 8:**
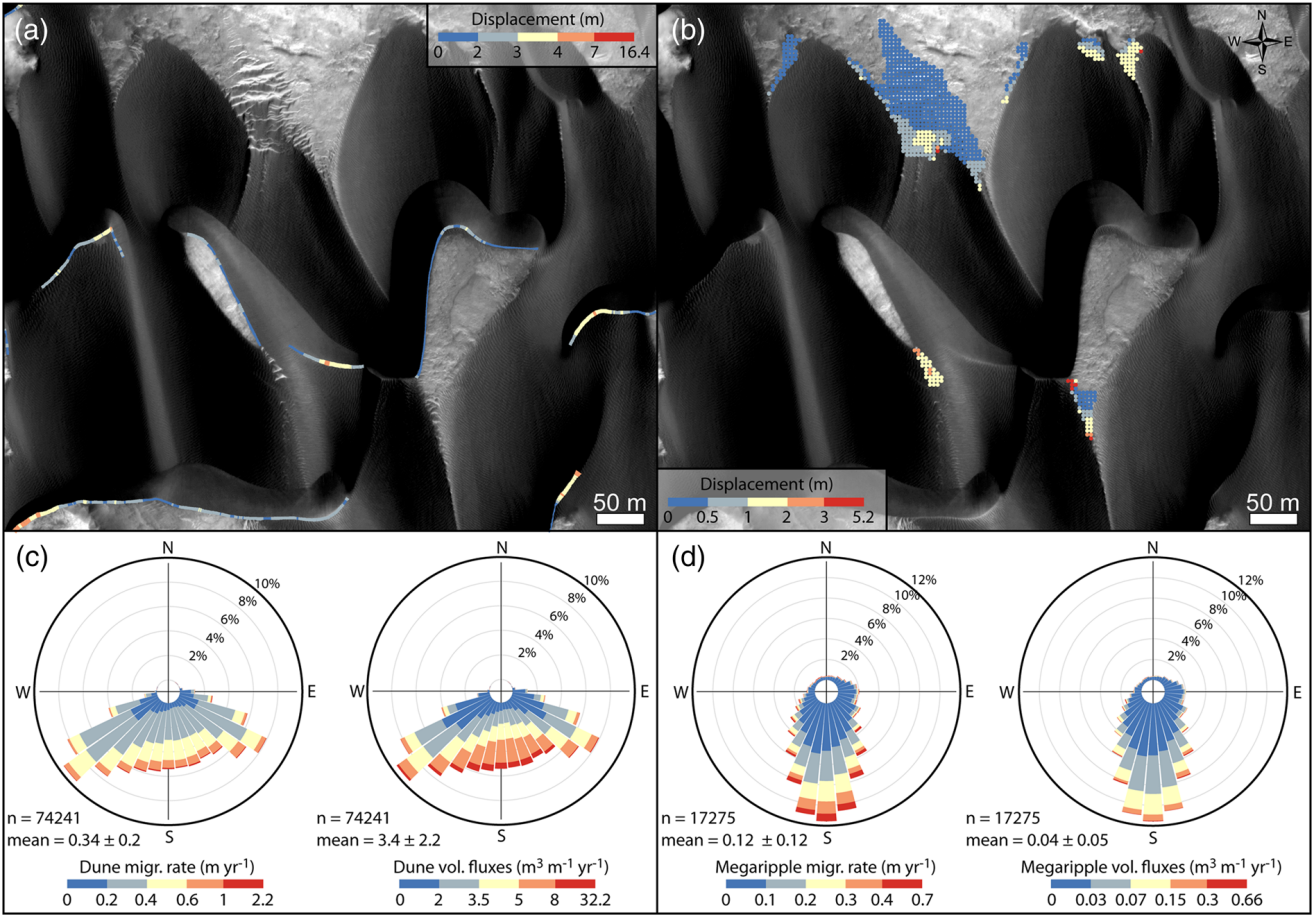
Bedform migration in McLaughlin crater. (a) Dune displacement computation. (b) COSI Corr megaripple displacement. (c) Circular plot showing the distribution of the dune migration vectors (rates and fluxes) shown in panel (a). (d) Circular plot showing the distribution of the megaripple rate and flux vectors. (a, b) HiRISE ESP_045312_2020.

**Figure 9:**
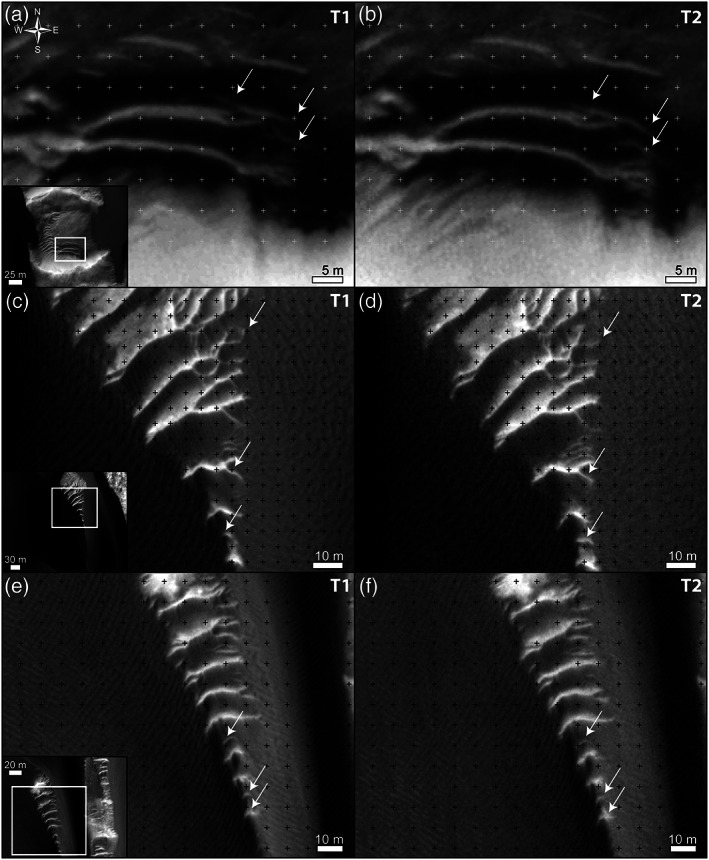
Bright‐toned megaripple (small TARs) migration in McLaughlin crater. White arrows indicate the same ripple in the two subimages at T1 and T2 (Table 1). Note how the megaripple position changes compared to the reference grid. Context on bottom‐left insets. (a and b) Migration of megaripple defects surrounded by active dark sand inside an ~130‐km‐wide impact crater (same areas in Animations S10 and S11). Grid spacing is 5 m. (c and d) Megaripple defect migration in between two dunes. Also the smaller (shorter‐wavelength) megaripples are moving (Animation S13). Grid spacing is 8 m. (e and f) Only the smaller (less spaced) elements of the megaripple fields are moving (Animation S14). Grid spacing is 10 m.

Despite rare exceptions (Animation S6), the megaripple crests and dune lee sides' share similar orientations and displacement directions, which suggest similar wind patterns (Figures 7a inset and 7d). TARs and megaripples migrated toward the south during the T1‐T2 time span in agreement with their main EW crest orientations (Figures 8 and 9 and Animations S5 and S7–S14). Locally, longitudinal megaripple migration can be seen (Animation S6). Dunes moved on average 2.6 m toward the S (0.34 m yr^−1^) while megaripples moved 0.9 m (0.12 m yr^−1^) (Figure 8 and Table 2). On average, megaripples in McLaughlin crater migrated ~3 time slower than dunes. Megaripple fluxes are, like in Nili Fossae, 2 orders of magnitude lower than dune fluxes (3.4 and 0.04 m^3^ m^−1^ yr^−1^ for dunes and megaripple, respectively) (Figures 8c and 8d and Table 2). In McLaughlin crater, even isolated bright‐toned megaripple patches located in impact craters are active (Figure 9a and Animations S10 and S11). This is a surprising result as these areas are lower in the boundary layer and would be subject to less wind. In some cases, only the ripple defects (terminations) that are proximal or directly in contact with active dark sand are moving (Figures 9a–9d and Animations S10–S12). In other areas, part of the megaripple train is static, but the migration of the smaller (shorter‐wavelength) elements suggest that even the larger features should be active in the long term (Figures 9c–9f and Animations S13 and S14). Note the high albedo of the megaripples shown in Figure 9—these bedforms have a clear resemblance to TARs (Fenton et al., 2003; Zimbelman, 2010).

## 4 Discussion

### 4.1 Megaripple Migration and Fluxes

In this paper, we present the first quantification of megaripple movement on Mars and show unambiguous evidence for bright‐toned megaripple (or TAR) migration. Zimbelman (2019) differentiate megaripples and small TARs on the base of their albedo and wavelength, where the latter are brighter and have larger spacing. Although, only a proper and widespread analysis of bedforms wavelength on Mars can help to highlight differences (if present) between megaripples and TARs, our results show that not all the bright‐toned bedforms on Mars are relict of a past climate. As already suggested to explain the higher albedo of similar bedforms elsewhere on Mars, the bright‐toned appearance of active megaripples in McLaughlin crater could be due to the accumulation of dust over slowly moving features such as the study megaripples (Silvestro et al., 2011; Zimbelman, 2019). Alternatively, a coarse‐grained component and/or a different grain composition compared to the nearby LRs could explain the higher albedo of megaripples.

**Figure 10:**
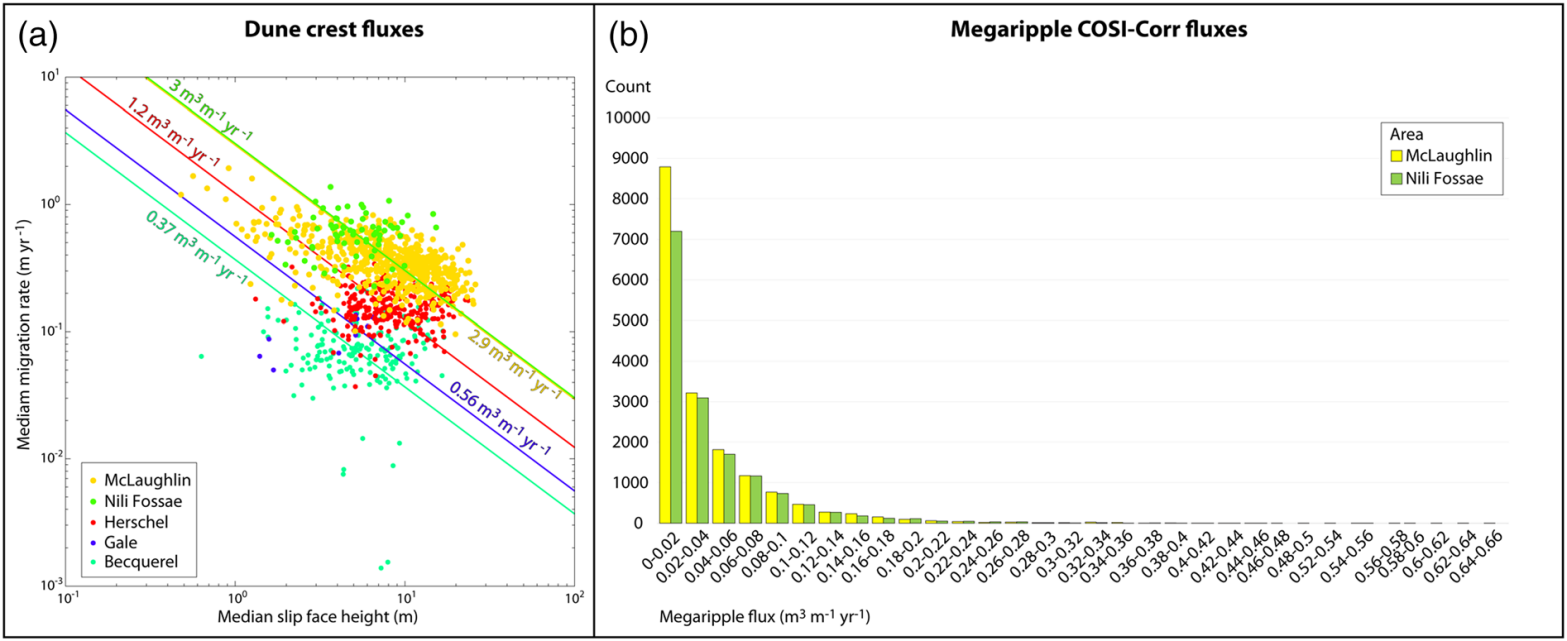
Dune and megaripple flux distributions. (a) Logarithmic plot of the dune median heights and respective median migration rate values for the two study areas and for other dune fields on Mars. Isoflux lines are also plotted. Note the similarity between Nili and Mclaughlin dune median fluxes and how these dunes have higher fluxes than other dunes on Mars. Bequerel values are computed from Urso et al. (2018), Gale values are computed using the same image analyzed in Silvestro et al. (2016a, 2016b), and Herschel values are computed from Cardinale et al. (2016) (see also Figure S5). (b) Megaripple flux distributions for the two study areas.

The origin and nature of certain aeolian bedforms on Mars, and even on Earth, is an open and debated question (Lämmel et al., 2018; Lapotre et al., 2016a; Sullivan & Kok, 2017; Vaz et al., 2017). For instance, dark‐toned LRs have been interpreted as fluid drag ripples with wavelength scaling with atmospheric kinematic viscosity (and in turn with atmospheric pressure) (Lapotre et al., 2016a, 2016b, 2018). However, despite being locally found in continuity with LRs (Ewing et al., 2017; Yizhaq et al., 2019; Zimbelman, 2019), Nili Fossae and McLaughlin TARs and megaripples at greater spacings and heights do not fit the fluid drag hypothesis (Lapotre et al., 2016a, 2018). Based on terrestrial studies, the coarse grains (>0.5 mm) accumulating on megaripples associated with dunes (not lying on bare rock surfaces) move by rolling and creep, and thus need smaller sand‐sized saltators to be displaced (Bagnold, 1954; Bridges et al., 2015; Kok et al., 2012; Yizhaq, Katra, Isenberg, & Tsoar, 2012). An ample supply of saltating sand within the interdune areas are suggested by the presence of dunes that maintain their shape while moving downwind. Looking at the plot in Figures 10a, we can see how dune fluxes in Nili Fossae and McLaughlin are between 5 and 7 times higher than the ones measured with the same method in Gale and Becquerel craters and ~3 times higher than the western Herschel dune fluxes (Figure S5) (Cardinale et al., 2016; Urso et al., 2018).

Such a relatively high volume of mobile sand is providing enough energy to the coarse particles to be displaced causing the megaripples to move at a rate detectable in the ~5 MY period of the study images. In the heavily imaged *Curiosity* landing site in Gale crater, where only limited evidence of coarse sediment (1–3 mm) was reported, megaripple migrations have not been detected so far (Bridges et al., 2017). We suggest the dune fluxes in Gale, with megaripples of similar wavelengths than Nili Fossae and McLaughlin crater, are not high enough (Figure 10a) to produce detectable megaripple displacements on subsequent HiRISE images. Conversely, the two study sites described here display relatively high dune fluxes compared with Gale and other areas on Mars (e.g., Herschel and Bequerel) (Bridges et al., 2017; Cardinale et al., 2016; Chojnacki et al., 2018, 2019; Silvestro et al., 2016a; Urso et al., 2018) (Figure 10a), suggesting that megaripple migration reported herein was driven by impact‐driven creep as the dominant process. In addition, the dune crest fluxes in the study areas have similar distributions and medians as highlighted in Figure 10a and in Table 2. Thus, with similar dune fluxes and in turn a similar amount of saltators available in Nili Fossae and McLaughlin, we would expect comparable megaripple fluxes in the two study areas, which have megaripples with comparable sizes (5–35 m) (Figure 4). Such a similarity is evident in Figure 10b reinforcing the existence of a relationship between dune migration/fluxes and megaripple migration/fluxes at the dune field scale. Thus, our results support previous interpretations of megaripples/TARs as analogs to terrestrial megaripples where coarse grains are displaced by rolling/creep caused by saltating sand impactors (Foroutan et al., 2018; Hugenholtz et al., 2017; Hugenholtz & Barchyn, 2017; Lapotre et al., 2016a). This coupling between active sand and megaripple migration seems to be valid even locally, where only the megaripple portions that are directly in contact with the dune sand are moving (Figure 9 and Animations S10 and S11). In addition, our findings suggest that winds strong enough to saltate fine to medium sand‐sized grains, abundant on Martian dunes (Charles et al., 2016; Weitz et al., 2018), would be sufficient to displace the coarse grains accumulating on the megaripple crests, given an abundance of saltating sand. However, the wind conditions necessary to move and maintain the megaripples are not constrained at the two study sites. Even at Gale crater only localized motion of coarse grains was detected by *Curiosity*, while megaripples appeared static (Baker et al., 2018; Bridges et al., 2017). Terrestrial studies indicate that in general megaripples are formed and shaped by strong winds (Lorenz & Valdez, 2011; Sharp, 1963) and that gusts of sufficient strength to move these bedforms are highly energetic but intermittent, often associated with rare storm events (Isenberg et al., 2011; Milana, 2009; Sakamoto‐Arnold, 1981; Yizhaq & Katra, 2015). Being the most resilient to movement among all aeolian bedforms (Hugenholtz & Barchyn, 2017; Sharp, 1963), finding megaripples migrating on Mars is surprising due to the low density of the present‐day atmosphere and the intermittency of sand‐moving winds (Day & Rebolledo, 2019), especially in locations shielded by strong winds such as craters (Figures 7b, 9a, and 9b) or downwind of tall dune lee faces (Animations S6 and S7). A better understanding of the Martian atmosphere through modeling (even at high spatial scale, Jackson et al., 2015) and more in situ wind measurements are still needed to explain the findings of this work.

**Figure 11:**
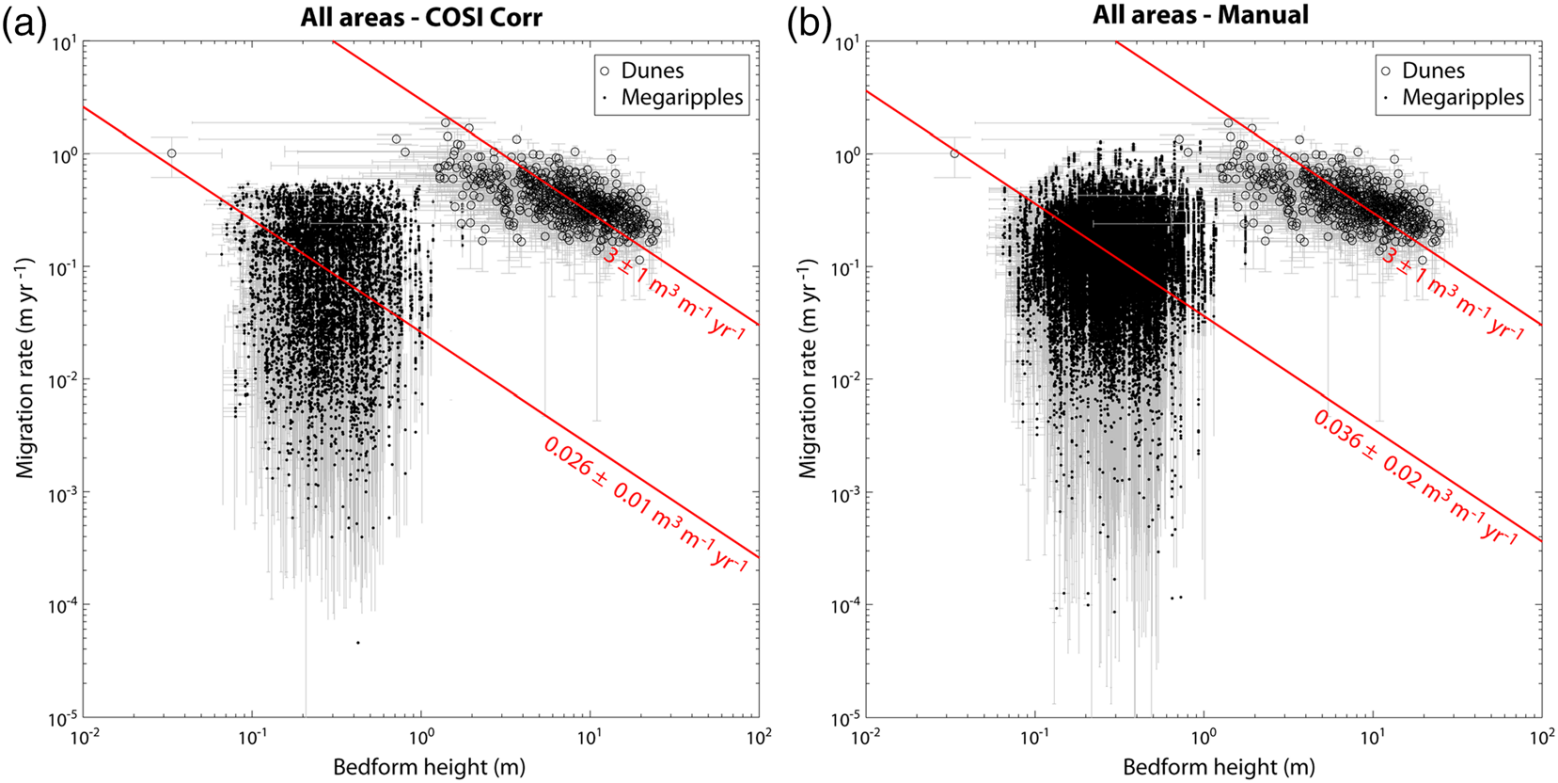
Dune and megaripple migration rate comparison for COSI‐Corr (a) and manual (b) measurements in the two study areas. The megaripple fluxes are 2 orders of magnitude lower than dune fluxes (red isoflux lines). Note the similarity between the COSI‐Corr and manual measurements (see also Figure S6).

Our results also show that the moving megaripples have fluxes that are 2 orders of magnitude lower than dune fluxes on average as highlighted in Figure 11. The similarity between COSI‐Corr (Figure 11a) and manual megaripple flux measurements (Figures 11b and S6) highlights the robustness of our analysis (see also Table 2). A low megaripple flux is expected due to the presumed different nature of the transport for dunes and megaripples, namely, saltation/reptation for dunes (Bridges et al., 2012a) and impact creep for the megaripples and LRs. LRs located on the dune slopes likely have higher fluxes than megaripples based on their swifter migration rates. Unfortunately, LR fluxes cannot be computed in the study areas using the Table 1 images as these features moved too fast to be confidently tracked with COSI‐Corr. When compared to LR fluxes measured in other areas of Mars, we notice that the megaripple fluxes measured in this work are 2 orders of magnitude lower than LR fluxes measured in Nili Patera (Bridges et al., 2012a).

### 4.2 Megaripple Dynamic

In general, the megaripples we have analyzed in the two study areas show crestline orientations and vectorial migration azimuths consistent with dunes. Exceptions may occur with more complex megaripple morphologies, such as star‐shaped megaripple morphologies radially arranged around dunes (Animation S3) and megaripples displaying oblique and longitudinal migrations visible in the COSI‐Corr displacement maps (Animations S3, S4, and S6). Thus, from a dynamic point of view, Martian megaripples in the study areas are similar to LRs observed elsewhere on Mars and their flux might be slightly underestimated as we are missing the oblique/longitudinal flux component (Runyon, Bridges, Ayoub, et al., 2017; Silvestro et al., 2016a; Vaz et al., 2017). The intermittency of wind gusts above the threshold (Camola et al., 2019; Day & Rebolledo, 2019), together with the large dimension of the Martian ripples, is proposed reasons to explain oblique and longitudinal migration of Martian LRs (Silvestro et al., 2016a). This explanation should be even more valid for megaripples, which we have demonstrated to move much slower than LRs and can thus integrate a more complex wind signal (Ewing et al., 2014; Silvestro et al., 2016a).

Nontransverse megaripple orientations were observed on Earth in the Kumtag Desert of China close to topographic obstacles such as yardangs (Qian et al., 2012). However, megaripple dynamics and orientations compared to primary winds were not documented (Qian et al., 2012). Even long‐term (months) time‐lapse observations of megaripples in Egypt do not shown any oblique or longitudinal migration for ~30‐cm‐spaced megaripples located over a saddle between two seif dunes (Lorenz, 2011). However, time‐lapse monitoring of aeolian bedforms is still at its infancy. The results of this paper can boost the research on the dynamical behavior of megaripples on Earth and help to interpret analogous Martian features in terms of formative winds. This is key for a better understanding of the present climate on Mars and an improved interpretation of sandstone stratification in terms of paleo–flow directions.

## 5 Conclusions

In this work, we report and quantify the migration and sand fluxes of bright‐toned megaripples on Mars. To our knowledge, this is the first report of activity for this common class of aeolian bedforms including small TAR‐like landforms, which have previously been interpreted as dormant based on the lack of detectable motion and other evidence (e.g., super position of craters and fractures) (Banks et al., 2018; Berman et al., 2018; Bridges, Bourke, et al., 2012; Chojnacki et al., 2018; Silvestro et al., 2010; Sullivan et al., 2008). The evidence for megaripple migration reported here indicates that not all the bright‐toned megaripples on Mars are relict of a past climate.

This study also found active megaripples are most readily identified in association with high‐flux dunes, implying a relationship between dune and megaripple fluxes. This offers a prediction that can be tested for other known high flux dune sites (Chojnacki et al., 2019), which may host other active megaripples. The close spatial correlation between the two bedform classes also suggests that, similarly to terrestrial megaripples, impact creep is the main transport process for megaripple formation and movement, although confirmation of an exact grain transport mode must await in situ observations.

The computed average and median fluxes for megaripples are 2 orders of magnitude lower than dune fluxes, likely reflecting the different mode of aeolian transport for megaripples and dunes. It is also prudent to acknowledge that this type of detection was facilitated in part by the existence of long‐temporal baseline (~9 EYs and 5 MYs) repeat data sets such as NASA's MRO/HiRISE mission.

Locally, megaripples show oblique and longitudinal migration like the LRs over the dune slopes elsewhere on Mars. This might reflect the capability for this type of bedform to incorporate a complex wind signal and may be the result of the low gravity/density Martian atmospheric environment where sand transporting events are rare compared to Earth. Future studies, potentially inspired by this work, might shed light on the reasons that oblique and longitudinal megaripple migration is not found on Earth.

Finally, by extending the known size range of migrating aeolian bedforms in the contemporary Martian climate regime, the results reported here provide additional constraints for atmospheric and sand transport modeling efforts, whose improvement represent key steps toward a better comprehension of the climate of Mars.

## Supporting information



Supporting Information S1

Table S1

Movie S1

Movie S2

Movie S3

Movie S4

Movie S5

Movie S6

Movie S7

Movie S8

Movie S9

Movie S10

Movie S11

Movie S12

Movie S13

Movie S14

## Data Availability

Supporting information is available in the online version of the paper, including supplemental figures and animated GIFs. The data used for this investigation can be found at the HiRISE website (http://hirise.lpl.arizona.edu/, see Table S1) or the Planetary Data System (http://pds.nasa.gov/).

## References

[jgre21421-bib-0001] Ayoub, F. , Avouac, J. , Newman, C. E. , Richardson, M. I. , Lucas, A. , Leprince, S. , & Bridges, N. T. (2014). Threshold for sand mobility on Mars calibrated from seasonal variations of sand flux. Nature Communications, 5(1), 5096 10.1038/ncomms6096 25268931

[jgre21421-bib-0002] Bagnold, R. A. (1954). The physics of blown sand and desert dunes (2nd ed.). Mineola, New York: Dover publications, INC.

[jgre21421-bib-0003] Baker, M. M. , Newman, C. E. , Lapotre, M. G. A. , Sullivan, R. , Bridges, N. T. , & Lewis, K. W. (2018). Coarse sediment transport in the modern Martian environment. Journal of Geophysical Research: Planets, 123, 1380–1394. 10.1002/2017JE005513

[jgre21421-bib-0004] Balme, M. , Berman, D. C. , Bourke, M. C. , & Zimbelman, J. R. (2008). Transverse Aeolian Ridges (TARs) on Mars. Geomorphology, 101(4), 703–720. 10.1016/j.geomorph.2008.03.011

[jgre21421-bib-0005] Banks, M. E. , Fenton, L. K. , Bridges, N. T. , Geissler, P. E. , Chojnacki, M. , Runyon, K. D. , Silvestro, S. , & Zimbelman, J. R. (2018). Patterns in mobility and modification of middle‐ and high‐latitude Southern Hemisphere dunes on Mars. Journal of Geophysical Research: Planets, 123, 3205–3219. 10.1029/2018JE005747

[jgre21421-bib-0006] Berman, D. C. , Balme, M. R. , Michalski, J. R. , Clark, S. C. , & Joseph, E. C. S. (2018). High‐resolution investigations of Transverse Aeolian Ridges on Mars. Icarus, 312, 247–266. 10.1016/j.icarus.2018.05.003

[jgre21421-bib-0007] Berman, D. C. , Balme, M. R. , Rafkin, S. C. R. , & Zimbelman, J. R. (2011). Transverse Aeolian Ridges (TARs) on Mars II: Distributions, orientations, and ages. Icarus, 213(1), 116–130. 10.1016/j.icarus.2011.02.014

[jgre21421-bib-0008] Bhardwaj, A. , Sam, L. , Martin‐torres, F. J. , & Zorzano, M. (2019). Distribution and morphologies of transverse Aeolian ridges in ExoMars 2020 Rover landing site. Remote Sensing, 11(8), 1–17. 10.3390/rs11080912

[jgre21421-bib-0009] Bourke, M. C. , Lancaster, N. , Fenton, L. K. , Parteli, E. J. R. , Zimbelman, J. R. , & Radebaugh, J. (2010). Extraterrestrial dunes: An introduction to the special issue on planetary dune systems. Geomorphology, 121(1–2), 1–14. 10.1016/j.geomorph.2010.04.007

[jgre21421-bib-0010] Bourke, M. C. , Wilson, S. A. , & Zimbelman, J. R. (2003). The variability of transverse aeolian ridges in troughs on Mars In Lunar and Planetary Science XXXIV (pp. 2090). Lunar and Planetary Institute (LPI) https://www.lpi.usra.edu/meetings/lpsc2003/pdf/2090.pdf, https://ntrs.nasa.gov/search.jsp?R=20030111360

[jgre21421-bib-0011] Bridges, N. , Geissler, P. , Silvestro, S. , & Banks, M. (2013). Bedform migration on Mars: Current results and future plans. Aeolian Research, 9, 133–151. 10.1016/j.aeolia.2013.02.004

[jgre21421-bib-0012] Bridges, N. T. , Ayoub, F. , Avouac, J.‐P. , Leprince, S. , Lucas, A. , & Mattson, S. (2012a). Earth‐like sand fluxes on Mars. Nature, 485(7398), 339–342. 10.1038/nature11022 22596156

[jgre21421-bib-0013] Bridges, N. T. , Ayoub, F. , Avouac, J.‐P. , Leprince, S. , Lucas, A. , & Mattson, S. (2012b). Earth‐like sand fluxes on Mars. Supplementary Information. Nature, 485(7398), 339–342. 10.1038/nature11022 22596156

[jgre21421-bib-0014] Bridges, N. T. , Bourke, M. C. , Geissler, P. E. , Banks, M. E. , Colon, C. , Diniega, S. , Golombek, M. P. , Hansen, C. J. , Mattson, S. , McEwen, A. S. , Mellon, M. T. , Stantzos, N. , & Thomson, B. J. (2012). Planet‐wide sand motion on Mars. Geology, 40(1), 31–34. 10.1130/G32373.1

[jgre21421-bib-0015] Bridges, N. T. , Geissler, P. E. , McEwen, A. S. , Thomson, B. J. , Chuang, F. C. , Herkenhoff, K. E. , Keszthelyi, L. P. , & Martínez‐Alonso, S. (2007). Windy Mars: A dynamic planet as seen by the HiRISE camera. Geophysical Research Letters, 34, L23205 10.1029/2007GL031445

[jgre21421-bib-0016] Bridges, N. T. , Spagnuolo, M. G. , de Silva, S. L. , Zimbelman, J. R. , & Neely, E. M. (2015). Formation of gravel‐mantled megaripples on Earth and Mars: Insights from the Argentinean Puna and wind tunnel experiments. Aeolian Research, 17, 49–60. 10.1016/j.aeolia.2015.01.007

[jgre21421-bib-0017] Bridges, N. T. , Sullivan, R. , Newman, C. E. , Navarro, S. , van Beek, J. , Ewing, R. C. , Ayoub, F. , Silvestro, S. , Gasnault, O. , le Mouélic, S. , Lapotre, M. G. A. , & Rapin, W. (2017). Martian aeolian activity at the Bagnold Dunes, Gale Crater: The view from the surface and orbit. Journal of Geophysical Research: Planets, 122, 2077–2110. 10.1002/2017JE005263

[jgre21421-bib-0018] Camola, F. , Kok, J. F. , Chamecki, M. , & Martin, R. L. (2019). The intermittency of wind‐driven sand transport. Geophysical Research Letters, 46, 1–11. 10.1029/2019GL085739/2019GL085739

[jgre21421-bib-0019] Cardinale, M. , Silvestro, S. , Vaz, D. A. , Michaels, T. , Bourke, M. C. , Komatsu, G. , & Marinangeli, L. (2016). Present‐day aeolian activity in Herschel crater, Mars. Icarus, 265, 139–148. 10.1016/j.icarus.2015.10.022

[jgre21421-bib-0020] Charles, H. , Titus, T. , Hayward, R. , Edwards, C. , & Ahrens, C. (2016). Comparison of the mineral composition of the sediment found in two Mars dunefields: Ogygis Undae and Gale crater—Three distinct endmembers identified. Earth and Planetary Science Letters, 458, 152–160. 10.1016/j.epsl.2016.10.022

[jgre21421-bib-0021] Chojnacki, M. , Banks, M. , & Urso, A. (2018). Wind‐driven erosion and exposure potential at Mars 2020 Rover candidate‐landing sites. Journal of Geophysical Research: Planets, 123, 468–488. 10.1002/2017JE005460 29568719 PMC5859260

[jgre21421-bib-0022] Chojnacki, M. , Banks, M. E. , Fenton, L. K. , & Urso, A. C. (2019). Boundary condition controls on the high‐sand‐flux regions of Mars. Geology, 47(5), 1–4. 10.1130/G45793.1/4657361/g45793.pdf PMC7241575 32440031

[jgre21421-bib-0023] Chojnacki, M. , Johnson, J. R. , Moersch, J. E. , Fenton, L. K. , Michaels, T. I. , & Bell, J. F. (2014). Persistent aeolian activity at Endeavour crater, Meridiani Planum, Mars; new observations from orbit and the surface. Icarus, 251, 275–290. 10.1016/j.icarus.2014.04.044

[jgre21421-bib-0024] Davis, J. M. , Grindrod, P. M. , Boazman, S. J. , Vermeesch, P. , & Baird, T. (2019). Quantified Aeolian dune changes on Mars derived from repeat Context Camera images. Earth and Space Science, 7(2). 10.1029/2019EA000874

[jgre21421-bib-0025] Day, M. , & Rebolledo, L. (2019). Intermittency in wind‐driven surface alteration on Mars interpreted from wind streaks and measurements by InSight. Geophysical Research Letters, 46, 12,747–12,755. 10.1029/2019GL085178

[jgre21421-bib-0026] Dickinson, J. L. , Kerber, L. A. , Fasset, C. I. , & Ehlmann, B. L. (2018). A global, blended CTX mosaic of Mars with vectorized seam mapping: A new mosaicking pipeline using principles of non‐destructive image editing In 49th Lunar and Planetary Science Conference (Vol. 49, pp. 1–2). Lunar and Planetary Institute (LPI) https://www.hou.usra.edu/meetings/lpsc2018/pdf/2480.pdf, https://ui.adsabs.harvard.edu/abs/2018LPI....49.2480D/abstract

[jgre21421-bib-0027] Diniega, S. , Krevalevsky, M. , Radebaugh, J. , Silverstro, S. , Telfer, M. , & Tirsch, D. (2017). Our evolving understanding of aeolian bedforms, based on observation of dunes on different worlds. Aeolian Research, 26, 5–27. 10.1016/j.aeolia.2016.10.001

[jgre21421-bib-0028] Edgett, K. , & Malin, M. (2000). New views of Mars eolian activity, materials, and surface properties: Three vignettes from the Mars Global Surveyor Mars Orbiter camera. Journal of Geophysical Research, 105(E1), 1623–1650. 10.1029/1999JE001152

[jgre21421-bib-0029] Ewing, R. C. , Lapotre, M. G. A. , Lewis, K. W. , Day, M. , Stein, N. , Rubin, D. M. , Sullivan, R. , Banham, S. , Lamb, M. P. , Bridges, N. T. , Gupta, S. , & Fischer, W. W. (2017). Sedimentary processes of the Bagnold Dunes: Implications for the eolian rock record of Mars. Journal of Geophysical Research: Planets, 122, 2544–2573. 10.1002/2017JE005324 29497590 PMC5815379

[jgre21421-bib-0030] Ewing, R. C. , McDonald, G. D. , & Hayes, A. G. (2014). Multi‐spatial analysis of aeolian dune‐field patterns. Geomorphology, 240, 44–53. 10.1016/j.geomorph.2014.11.023

[jgre21421-bib-0031] Fenton, L. K. (2005). Aeolian processes in Proctor Crater on Mars: Mesoscale modeling of dune‐forming winds. Journal of Geophysical Research, 110, E06005 10.1029/2004JE002309

[jgre21421-bib-0032] Fenton, L. K. , Bandfield, J. L. , & Ward, A. W. (2003). Aeolian processes in Proctor Crater on Mars: Sedimentary history as analyzed from multiple data sets. Journal of Geophysical Research, 108(E12), 5129 10.1029/2002JE002015

[jgre21421-bib-0033] Foroutan, M. , Steinmetz, G. , Zimbelman, J. R. , & Duguay, C. R. (2018). Megaripples at Wau‐an‐Namus, Libya: A new analog for similar features on Mars. Icarus, 319(October 2018), 840–851. 10.1016/j.icarus.2018.10.021

[jgre21421-bib-0034] Geissler, P. E. (2014). The birth and death of transverse aeolian ridges on Mars. Journal of Geophysical Research: Planets, 119, 2583–2599. 10.1002/2014JE004633

[jgre21421-bib-0035] Geissler, P. E. , Stantzos, N. W. , Bridges, N. T. , Bourke, M. C. , Silvestro, S. , & Fenton, L. K. (2012). Shifting sands on Mars: Insights from tropical intra‐crater dunes. Earth Surface Processes and Landforms, 38(4), 407–412. 10.1002/esp.3331

[jgre21421-bib-0036] Geissler, P. E. , & Wilgus, J. T. (2017). The morphology of transverse aeolian ridges on Mars. Aeolian Research, 119(12), 2583–2599. 10.1002/2014JE004633

[jgre21421-bib-0037] Greeley, R. , & Iversen, J. (1985). Wind as a geological process on Earth, Mars, Venus and Titan, Cambridge, UK: Cambridge University Press 10.1017/CBO9780511573071

[jgre21421-bib-0038] Hugenholtz, C. H. , & Barchyn, T. E. (2017). A terrestrial analog for transverse aeolian ridges (TARs): Environment, morphometry, and recent dynamics. Icarus, 289, 239–253. 10.1016/j.icarus.2016.08.010

[jgre21421-bib-0039] Hugenholtz, C. H. , Barchyn, T. E. , & Boulding, A. (2017). Morphology of transverse aeolian ridges (TARs) on Mars from a large sample: Further evidence of a megaripple origin? Icarus, 286, 193–201. 10.1016/j.icarus.2016.10.015

[jgre21421-bib-0040] Isenberg, O. , Yizhaq, H. , Tsoar, H. , Wenkart, R. , Karnieli, A. , Kok, J. F. , & Katra, I. (2011). Megaripple flattening due to strong winds. Geomorphology, 131(3–4), 69–84. 10.1016/j.geomorph.2011.04.028

[jgre21421-bib-0041] Jackson, D. W. T. , Bourke, M. C. , & Smyth, T. A. G. (2015). The dune effect on sand‐transporting winds on Mars. Nature Communications, 6(1), 8796 10.1038/ncomms9796 PMC4667610 26537669

[jgre21421-bib-0042] Kerber, L. , & Head, J. W. (2012). A progression of induration in Medusae Fossae Formation transverse aeolian ridges: Evidence for ancient aeolian bedforms and extensive reworking. Earth Surface Processes and Landforms, 37(4), 422–433. 10.1002/esp.2259

[jgre21421-bib-0043] Kirk, R. L. , Howington‐Kraus, E. , Rosiek, M. R. , Anderson, J. A. , Archinal, B. A. , Becker, K. J. , Cook, D. A. , Galuszka, D. M. , Geissler, P. E. , Hare, T. M. , Holmberg, I. M. , Keszthelyi, L. P. , Redding, B. L. , Delamere, W. A. , Gallagher, D. , Chapel, J. D. , Eliason, E. M. , King, R. , & McEwen, A. S. (2008). Ultrahigh resolution topographic mapping of Mars with MRO HiRISE stereo images: Meter‐scale slopes of candidate Phoenix landing sites. Journal of Geophysical Research, 113, E00A24 10.1029/2007JE003000

[jgre21421-bib-0044] Kok, J. F. , Parteli, E. J. R. , Michaels, T. I. , & Karam, D. B. (2012). The physics of wind‐blown sand and dust. Reports on Progress in Physics, 75(10), 106901 10.1088/0034-4885/75/10/106901 22982806

[jgre21421-bib-0045] Lämmel, M. , Meiwald, A. , Yizhaq, H. , Tsoar, H. , Katra, I. , & Kroy, K. (2018). Aeolian sand sorting and megaripple formation. Nature Physics, 14(7), 759–765. 10.1038/s41567-018-0106-z

[jgre21421-bib-0046] Lancaster, N. (1995). Geomorphology of desert dunes. New York: Routledge.

[jgre21421-bib-0047] Lapotre, M. G. A. , Ewing, R. C. , Lamb, M. P. , Fischer, W. W. , Grotzinger, J. P. , Rubin, D. M. , Lewis, K. W. , Ballard, M. J. , Day, M. , Gupta, S. , Banham, S. G. , Bridges, N. T. , Marais, D. J. D. , Fraeman, A. A. , Grant, J. A. , Herkenhoff, K. E. , Ming, D. W. , Mischna, M. A. , Rice, M. S. , Sumner, D. Y. , Vasavada, A. R. , & Yingst, R. A. (2016a). Large wind ripples on Mars: A record of atmospheric evolution. Science *(80‐.)*, 353(6294), 55–58. 10.1126/science.aaf3206 27365444

[jgre21421-bib-0048] Lapotre, M. G. A. , Ewing, R. C. , Lamb, M. P. , Fischer, W. W. , Grotzinger, J. P. , Rubin, D. M. , Lewis, K. W. , Ballard, M. J. , Day, M. , Gupta, S. , Banham, S. G. , Bridges, N. T. , Marais, D. J. D. , Fraeman, A. A. , Grant, J. A. , Herkenhoff, K. E. , Ming, D. W. , Mischna, M. A. , Rice, M. S. , Sumner, D. Y. , Vasavada, A. R. , & Yingst, R. A. (2016b). Supplementary material: Large wind ripples on Mars: A record of atmospheric evolution. Science *(80‐.)*, 353(6294), 55–58. 10.1126/science.aaf3206 27365444

[jgre21421-bib-0049] Lapotre, M. G. A. , Ewing, R. C. , Weitz, C. M. , Lewis, K. W. , Lamb, M. P. , Ehlmann, B. L. , & Rubin, D. M. (2018). Morphologic diversity of Martian ripples: Implications for large‐ripple formation. Geophysical Research Letters, 45, 10,229–10,239. 10.1029/2018GL079029

[jgre21421-bib-0050] Leprince, S. , Barbot, S. , Ayoub, F. , & Avouac, J. P. (2007). Automatic, precise, ortho‐rectification and coregistration for satellite image correlation, application to ground deformation measurement. IEEE Transactions on Geoscience and Remote Sensing Letters, 45(6), 1529–1558. 10.1109/TGRS.2006.888937

[jgre21421-bib-0051] Lorenz, R. D. (2011). Observations of wind ripple migration on an Egyptian seif dune using an inexpensive digital timelapse camera. Aeolian Research, 3(2), 229–234. 10.1016/j.aeolia.2011.01.004

[jgre21421-bib-0052] Lorenz, R. D. , & Valdez, A. (2011). Variable wind ripple migration at Great Sand Dunes National Park and Preserve, observed by timelapse imaging. Geomorphology, 133(1–2), 1–10. 10.1016/j.geomorph.2011.06.003

[jgre21421-bib-0053] Malin, M. C. , Bell, J. F. III , Cantor, B. A. , Caplinger, M. A. , Calvin, W. M. , Clancy, R. T. , Edgett, K. S. , Edwards, L. , Haberle, R. M. , James, P. B. , Lee, S. W. , Ravine, M. A. , Thomas, P. C. , & Wolff, M. J. (2007). Context Camera Investigation on board the Mars Reconnaissance Orbiter. Journal of Geophysical Research, 112, E05S04 10.1029/2006JE002808

[jgre21421-bib-0054] Malin, M. C. , & Edgett, K. S. (2001). Mars Global Surveyor Mars Orbiter Camera: Interplanetary cruise through primary mission. Journal of Geophysical Research, 106(E10), 23,429–23,570. 10.1029/2000JE001455

[jgre21421-bib-0055] McEwen, A. S. , Eliason, E. M. , Bergstrom, J. W. , Bridges, N. T. , Hansen, C. J. , Delamere, W. A. , Grant, J. A. , Gulick, V. C. , Herkenhoff, K. E. , Keszthelyi, L. , Kirk, R. L. , Mellon, M. T. , Squyres, S. W. , Thomas, N. , & Weitz, C. M. (2007). Mars Reconnaissance Orbiter's High Resolution Imaging Science Experiment (HiRISE). Journal of Geophysical Research, 112, E05S02 10.1029/2005JE002605

[jgre21421-bib-0056] Milana, J. P. (2009). Largest wind ripples on earth? Geology, 37(4), 343–346. 10.1130/G25382A.1

[jgre21421-bib-0057] Pajola, M. , Rossato, S. , Baratti, E. , Pozzobon, R. , Quantin, C. , Carter, J. , & Thollot, P. (2017). Boulder abundances and size‐frequency distributions on Oxia Planum‐Mars: Scientific implications for the 2020 ESA ExoMars rover. Icarus, 296, 73–90. 10.1016/j.icarus.2017.05.011

[jgre21421-bib-0058] Qian, G. , Dong, Z. , Zhang, Z. , Luo, W. , & Lu, J. (2012). Granule ripples in the Kumtagh Desert, China: Morphology, grain size and influencing factors. Sedimentology, 59(6), 1888–1901. 10.1111/j.1365-3091.2012.01330.x

[jgre21421-bib-0059] Reiss, D. , van Gasselt, S. , Neukum, G. , & Jaumann, R. (2004). Absolute dune ages and implications for the time of formation of gullies in Nirgal Vallis, Mars. Journal of Geophysical Research, 109, E06007 10.1029/2004JE002251

[jgre21421-bib-0060] Runyon, K. D. , Bridges, N. T. , Ayoub, F. , Newman, C. E. , & Quade, J. J. (2017). An integrated model for dune morphology and sand fluxes on Mars. Earth and Planetary Science Letters, 457, 204–212. 10.1016/j.epsl.2016.09.054

[jgre21421-bib-0061] Runyon, K. D. , Bridges, N. T. , & Newman, C. E. (2017). Martian sand sheet characterization and implications for formation: A case study. Aeolian Research, 29(April), 1–11. 10.1016/j.aeolia.2017.09.001

[jgre21421-bib-0062] Sakamoto‐Arnold, C. M. (1981). Eolian features produced by the December 1977 windstorm, southern San Joaquin Valley, California. Journal of Geology, 54(1), 23–46. 10.1525/sp.2007.54.1.23

[jgre21421-bib-0063] Sharp, R. P. (1963). Wind ripples. Journal of Geology, 71(5), 617–636. 10.1086/626936

[jgre21421-bib-0064] Shockey, K. M. , & Zimbelman, J. R. (2012). Analysis of transverse aeolian ridge profiles derived from HiRISE images of Mars. Earth Surface Processes and Landforms, 38(2), 179–182. 10.1002/esp.3316

[jgre21421-bib-0065] Silvestro, S. , Fenton, L. K. , Vaz, D. A. , Bridges, N. T. , & Ori, G. G. (2010). Ripple migration and dune activity on Mars: Evidence for dynamic wind processes. Geophysical Research Letters, 37, L20203 10.1029/2010GL044743

[jgre21421-bib-0066] Silvestro, S. , Vaz, D. A. , Fenton, L. K. , & Geissler, P. E. (2011). Active aeolian processes on Mars: A regional study in Arabia and Meridiani Terrae. Geophysical Research Letters, 38, L20201 10.1029/2011GL048955

[jgre21421-bib-0067] Silvestro, S. , Vaz, D. A. , Yizhaq, H. , & Esposito, F. (2016a). Dune‐like dynamic of Martian Aeolian large ripples. Geophysical Research Letters, 43, 8384–8389. 10.1002/2016GL070014

[jgre21421-bib-0068] Silvestro, S. , Vaz, D. A. , Yizhaq, H. , & Esposito, F. (2016b). Supporting information: Dune‐like dynamic of Martian aeolian large ripples. Geophysical Research Letters, 43, 1–6. 10.1002/2016GL070014

[jgre21421-bib-0069] Siminovich, A. , Elperin, T. , Katra, I. , Kok, J. F. , Sullivan, R. , Silvestro, S. , & Yizhaq, H. (2019). Numerical study of shear stress distribution over sand ripples under terrestrial and Martian conditions. Journal of Geophysical Research: Planets, 124, 175–185. 10.1029/2018JE005701

[jgre21421-bib-0070] Sullivan, R. , Arvidson, R. , Bell, J. F. III , Gellert, R. , Golombek, M. , Greeley, R. , Herkenhoff, K. , Johnson, J. , Thompson, S. , Whelley, P. , & Wray, J. (2008). Wind‐driven particle mobility on Mars: Insights from Mars Exploration Rover observations at ‘“El Dorado”’ and surroundings at Gusev crater. Journal of Geophysical Research, 113, E06S07 10.1029/2008JE003101

[jgre21421-bib-0071] Sullivan, R. , & Kok, J. F. (2017). Aeolian saltation on Mars at low wind speeds. Journal of Geophysical Research: Planets, 122, 2111–2143. 10.1002/2017JE005275

[jgre21421-bib-0072] Sullivan, R. , Kok, J. F. , Yizhaq, H. , Siminovich, A. , Elperin, T. , & Katra, I. (2018). Low dynamic wind pressures on Mars allow a broad continuum of aeolian ripple sizes In X International Conference on Aeolian Research. https://colloque.inrae.fr/icar2018/content/download/4958/54415/version/1/file/Abstract_book_corrige_bis.pdf

[jgre21421-bib-0073] Urso, A. , Chojnacki, M. , & Vaz, D. A. (2018). Dune‐Yardang interactions in Becquerel crater, Mars. Journal of Geophysical Research: Planets, 123, 353–368. 10.1002/2017JE005465 29564199 PMC5857962

[jgre21421-bib-0074] Vaz, D. A. , Sarmento, P. T. K. , Barata, M. T. , Fenton, L. K. , & Michaels, T. I. (2015). Object‐based dune analysis: Automated dune mapping and pattern characterization for Ganges Chasma and Gale crater, Mars. Geomorphology, 250, 128–139. 10.1016/j.geomorph.2015.08.021

[jgre21421-bib-0075] Vaz, D. A. , & Silvestro, S. (2014). Mapping and characterization of small‐scale aeolian structures on Mars: An example from the MSL landing site in Gale crater. Icarus, 230, 151–161. 10.1016/j.icarus.2013.08.007

[jgre21421-bib-0076] Vaz, D. A. , Silvestro, S. , Sarmento, P. T. K. , & Cardinale, M. (2017). Migrating meter‐scale bedforms on Martian dark dunes: Are terrestrial aeolian ripples good analogues? Aeolian Research, 26, 101–116. 10.1016/j.aeolia.2016.08.003

[jgre21421-bib-0077] Vermeesch, P. , & Drake, N. (2008). Remotely sensed dune celerity and sand flux measurements of the world's fastest barchans (Bodele, Chad). Geophysical Research Letters, 35, L24404 10.1029/2008GL035921

[jgre21421-bib-0078] Weitz, C. M. , Sullivan, R. J. , Lapotre, M. G. A. , Rowland, S. K. , Grant, J. A. , Baker, M. , & Yingst, R. A. (2018). Sand grain sizes and shapes in aeolian bedforms at Gale crater, Mars. Geophysical Research Letters, 45, 9471–9479. 10.1029/2018GL078972

[jgre21421-bib-0079] Yizhaq, H. , Bel, G. , Silvestro, S. , Elperin, T. , Kok, J. F. , Cardinale, M. , Provenzale, A. , & Katra, I. (2019). The origin of the transverse instability of aeolian megaripples. Earth and Planetary Science Letters, 512, 59–70. 10.1016/j.epsl.2019.01.025

[jgre21421-bib-0080] Yizhaq, H. , & Katra, I. (2015). Longevity of aeolian megaripples, *Earth Planet* . Science Letters, 422, 28–32. 10.1016/j.epsl.2015.04.004

[jgre21421-bib-0081] Yizhaq, H. , Katra, I. , Isenberg, O. , & Tsoar, H. (2012). Evolution of megaripples from a flat bed. Aeolian Research, 6, 1–12. 10.1016/j.aeolia.2012.05.001

[jgre21421-bib-0082] Yizhaq, H. , Katra, I. , Kok, J. F. , & Isenberg, O. (2012). Transverse instability of megaripples. Geology, 40(5), 459–462. 10.1130/G32995.1

[jgre21421-bib-0083] Zimbelman, J. R. (2010). Transverse aeolian ridges on Mars: First results from HiRISE images. Geomorphology, 121(1–2), 22–29. 10.1016/j.geomorph.2009.05.012

[jgre21421-bib-0084] Zimbelman, J. R. (2019). The transition between sand ripples and megaripples on Mars. Icarus, 333(February), 127–129. 10.1016/j.icarus.2019.05.017

[jgre21421-bib-0085] Zimbelman, J. R. , Bourke, M. C. , & Lorenz, R. D. (2013). Recent developments in planetary Aeolian studies and their terrestrial analogs. Aeolian Research, 11, 109–126. 10.1016/j.aeolia.2013.04.004

[jgre21421-bib-0086] Zimbelman, J. R. , Irwin, R. P. , Williams, S. H. , Bunch, F. , Valdez, A. , & Stevens, S. (2009). The rate of granule ripple movement on Earth and Mars. Icarus, 203(1), 71–76. 10.1016/j.icarus.2009.03.033

