# Supplementary material for: Megaripple Migration on Mars
Source: J Geophys Res Planets. 2020 Jul 29;125(8):e2020JE006446. doi: 10.1029/2020JE006446 (PMC7583471; doi:10.1029/2020JE006446)
Supplement: Supplementary file 1 — Supporting Information S1 [file JGRE-125-e2020JE006446-s001.pdf]

AGU PUBLICATIONS

Journal of Geophysical Research – Planets

Supporting Information for

Megaripple Migration on Mars

S. Silvestro[1,2], M. Chojnacki[3,4], D. A. Vaz[5], M. Cardinale[6], H. Yizhaq[7], and F. Esposito[1]

[1]INAF Osservatorio Astronomico di Capodimonte, Via Moiariello 16, 80131, Napoli, Italia, [2]SETI Institute, Carl Sagan Center, 189 N. Bernardo Avenue, Suite 100, Mountain View, CA 94043, USA, [3]Lunar and Planetary Laboratory, University of Arizona, Tucson, AZ, USA, [4]Planetary Science Institute, Tucson, AZ, USA, [5]Centre for Earth and Space Research of the University of Coimbra, Observatório Geofísico e Astronómico da Universidade de Coimbra, Coimbra, Portugal, [6]DiSPUTer, Univ. G. d'Annunzio, Chieti, Italia, [7]Department of Solar Energy and Environmental Physics, BIDR, Ben-Gurion University of the Negev, Sede Boqer Campus 8499000, Israel

Corresponding author: Simone Silvestro (simone.silvestro@inaf.it)

Contents of this file

Figures S1 to S6 and captions
Supplemental animation captions
Supplemental Table S1

Additional Supporting Information (Files uploaded separately)

Animations S1-S14

Introduction

Supplementary material include the explanation of the method for computing the dune crest fluxes, together with one table (Table S1) and six images further complementing the method section in the main manuscript (Figures S1, S2, S5 and S6). The geographic context (Figures S3 and S4) for supplemental animations (Animations S1-S14) are also included. Animations show the migration of dunes and megaripples in the study areas, were produced in Photoshop and are fundamental for the comprehension of the paper.

Method for dune crest flux computation

All reported dune heights correspond to slip face brink heights. We use the semi-automatic procedure described in *Urso et al.* [2018] to derive the heights using the orthoimages and the DTM. This method uses the slip face base lines mapped from the different images to draw orthogonal profiles that are used to sample the image albedo in order to identify the high gradient areas, i.e. the brink areas. The DTM is used to impose geometric constrains on the location of the brink (slip face slopes = ~30º) and search for a curvature transitions that correspond to the slip face brink. The algorithm integrates all these parameters in order to automatically identify the location and elevation of the brink lines. Fluxes are computed by multiplying the heights and the displacements, generating continuous flux estimates along the slip faces. The method we use is slightly different from the ones used in other works [*Runyon et al.*, 2017; *Chojnacki et al.*, 2019], but should produce similar estimates.

Supplemental Figures

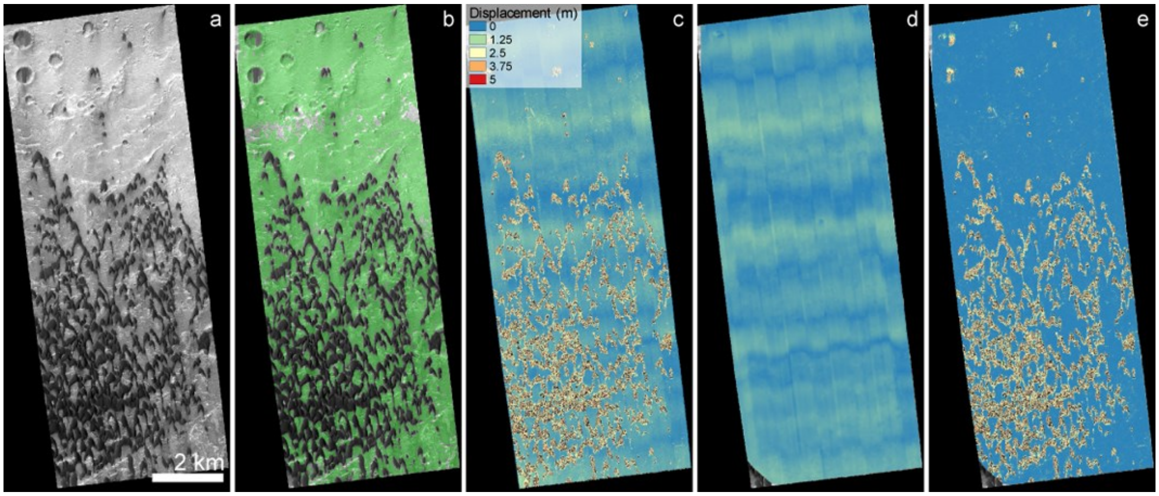

Figure S1. COSI-Corr jitter correction. (a) Nili Fossae HiRISE image. (b) Green areas correspond to bedrock areas identified by applying a threshold to the COSI-Corr signal-to-noise ratios. (c) Raw displacement map. (d) Jitter magnitude obtained by assuming zero displacement for the bedrock areas, jitter values for the dune covered areas are obtained by applying the bedrock mask, low pass filtering and inpainting. (e) Corrected displacement map, note that the EW and NS bands caused by jitter are no longer present.

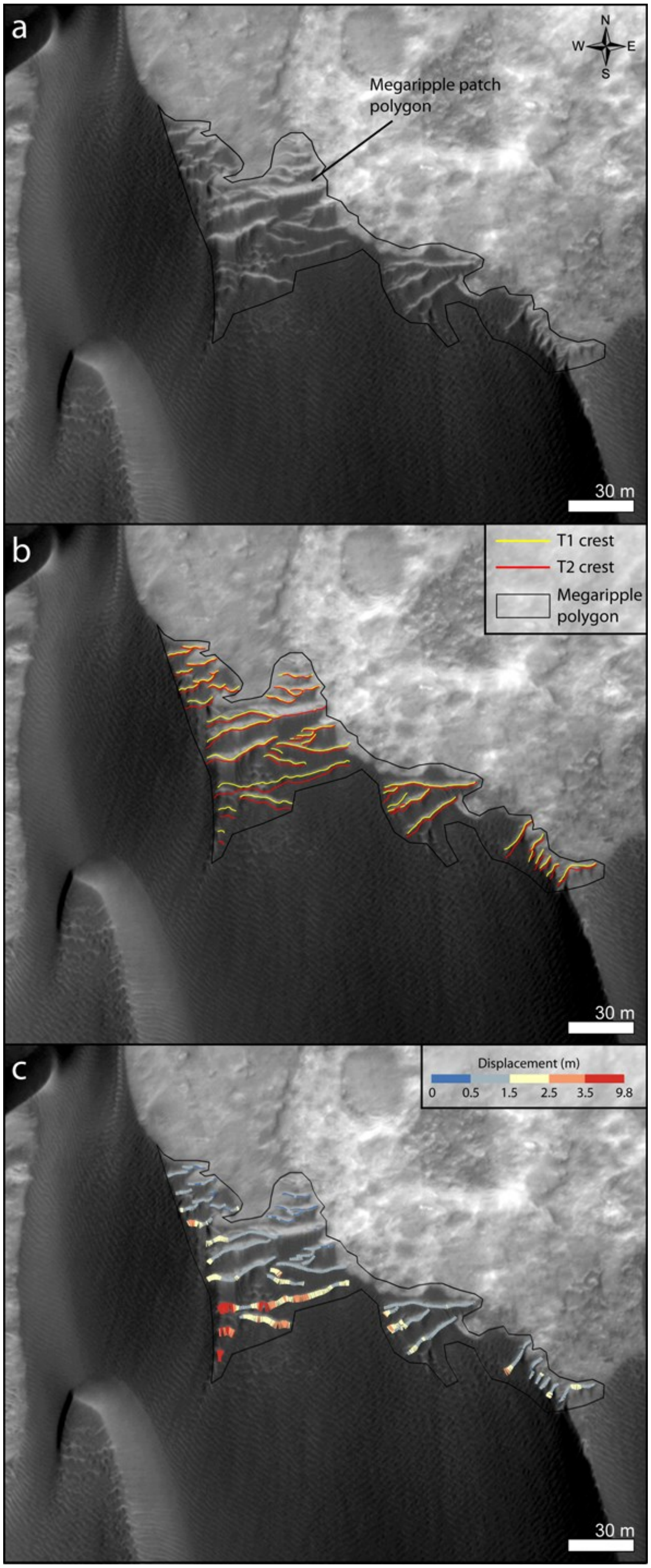

Figure S2. Manual mapping of the megaripples. (a) Megaripple locations are mapped in GIS. (b) Megaripple crestlines are mapped on the T1 and T2 images. (c) The perpendicular vectors connecting the megaripple crests are computed. HiRISE image ESP_045312_2020.

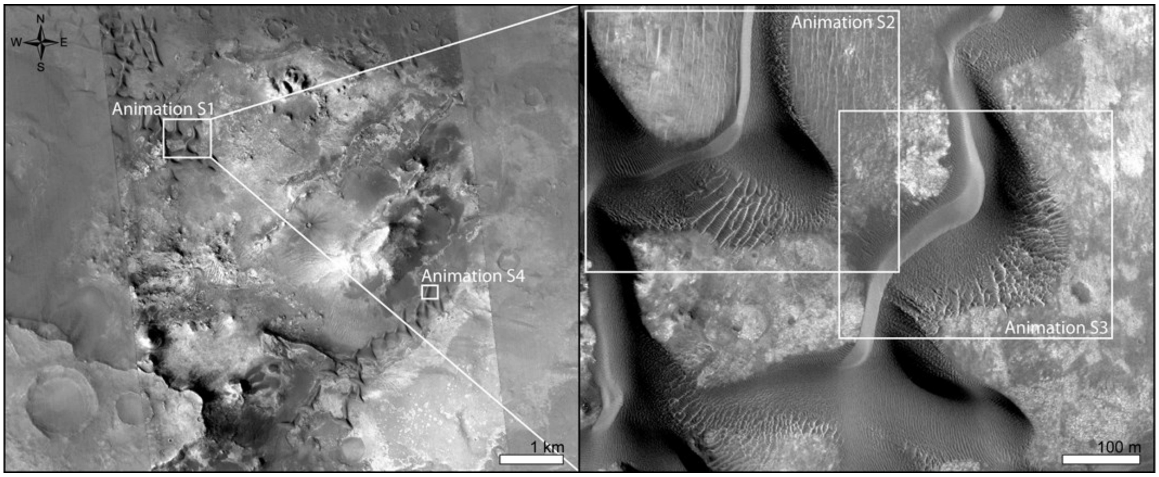

Figure S3. Geographical location for the Nili Fossae Animations S1-S4. HiRISE image ESP_047094_2015_RED.

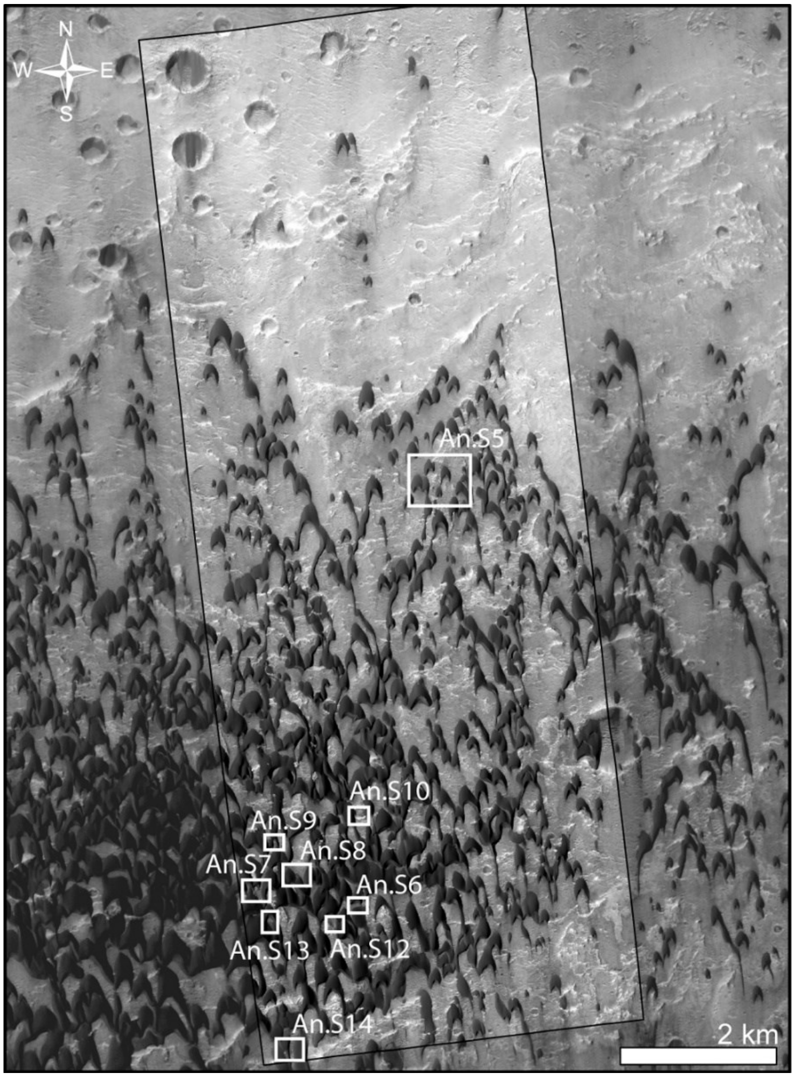

Figure S4. Geographical location for the McLaughlin Animations S5-S14. HiRISE image ESP_047094_2015_RED.

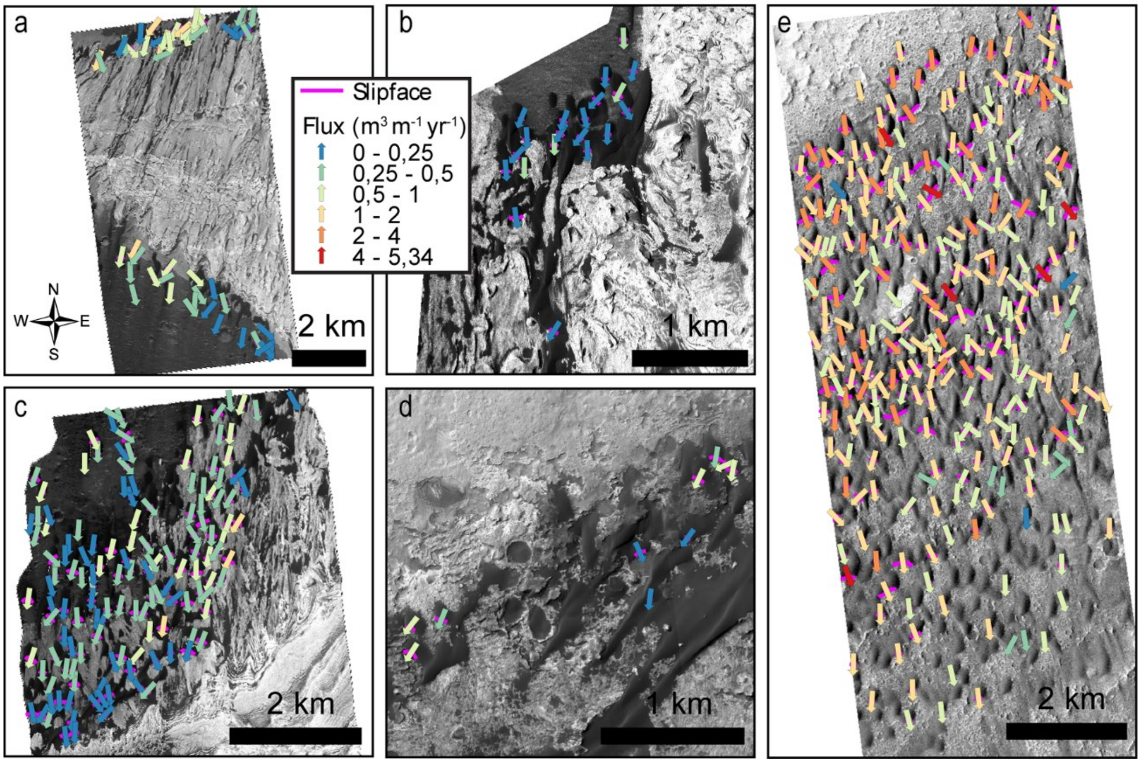

Figure S5. Crest flux computation for the additional areas of Figure 10a. (a-c) Becquerel crater, (d) Gale crater, (e) Herschel crater.

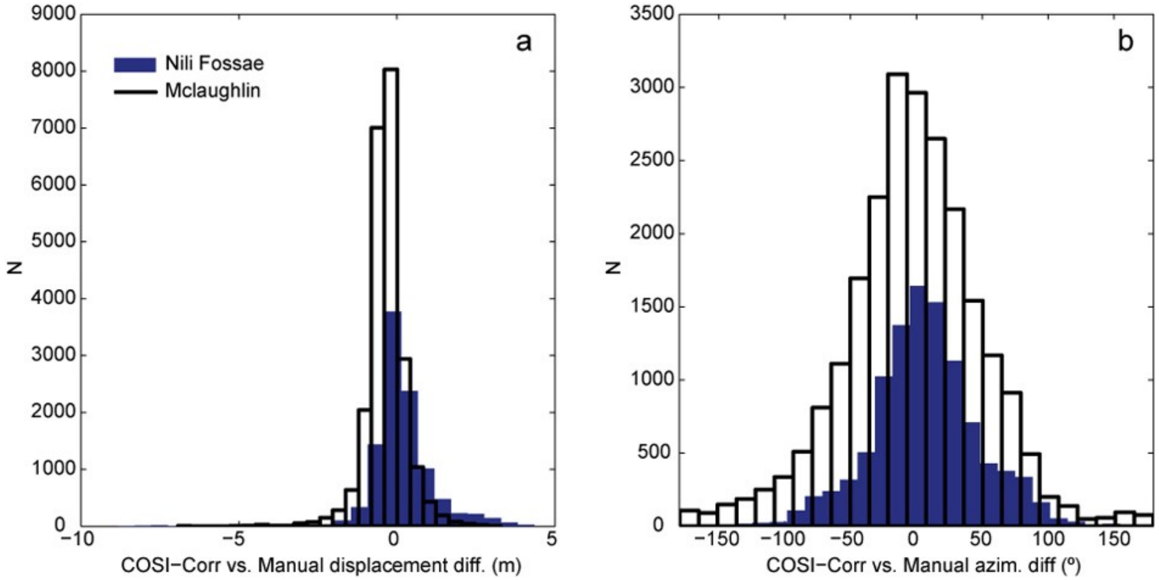

Figure S6. Comparison of measured displacement vectorial fields obtained with COSI-Corr and manual mapping for the selected megaripple areas. The COSI-Corr outputs were jitter corrected, while the bed forms that were manually mapped do not include this correction. (a) Histogram of the magnitude differences (the difference between COSI-Corr displacements and the displacement vectors obtained from the manual mapping and correlation of the bedform crestlines). We estimate average displacement differences of 0.18 ± 1.02 m and -0.34 ± 0.64 m for Nili Fossae and McLaughlin respectively. (b) The migration trends of the two datasets are also compatible, with average azimuth differences of 6.3 ± 41.0° and -2.9 ± 52.4° for Nili Fossae and McLaughlin respectively.

Supplemental Animation Captions
Animation S1. Migration of dunes and megaripples in Nili Fossae.
Animation S2. Migration of dunes and transverse megaripples in Nili Fossae.
Animation S3. Non-transverse (mostly oblique) migration of the megaripples in Nili Fossae. Note the complex (star-shaped) morphology of some megaripples and their radial orientation around the dune.
Animation S4. Elongation of longitudinal megaripples in Nili Fossae.
Animation S5. Migration of dunes and megaripples in McLaughlin crater.
Animation S6. Bright-toned longitudinal megaripples elongating behind the stoss side of a barchan dune in McLaughlin crater.
Animation S7. Migration of dunes and bright-toned megaripple in McLaughlin crater.
Animation S8. Migration of dunes and bright-toned megaripple in McLaughlin crater.
Animation S9. Migration of bright megaripple in McLaughlin crater. The moving megaripples are located stratigraphically below the dune.
Animation S10. Migration of bright megaripple defects inside a crater in McLaughlin. See also Animation S11.
Animation S11. Zoom on the migrating megaripple terminations of Animation S10. Only the crestline margins surrounded by the dark active sand are moving.
Animation S12. Migration of bright megaripple defects in McLaughlin crater.
Animation S13. Moving bright-toned megaripples in McLaughlin crater. Only the smaller (less spaced) megaripples and the crest terminations are moving.
Animation S14. Moving bright-toned megaripples in McLaughlin crater. Only the smaller (less spaced) megaripples and the crest terminations are moving.

Supplemental Tables

Nili Fossae			
**DTM ID**	**Left Observation**	**Right Observation**	**Web Link**
DTEEC_003587_2015_003086_2015_A01	PSP_003587_2015	PSP_003086_2015	https://www.uahirise.org/dtm/dtm.php?ID=PSP_003587_2015
DTEEC_045071_2015_045137_2015_U01	ESP_045071_2015	ESP_045137_2015	https://www.uahirise.org/dtm/dtm.php?ID=ESP_045071_2015
**McLaughlin crater**			
DTEEC_036859_2020_036569_2020_A01	ESP_036859_2020	ESP_036569_2020	https://www.uahirise.org/dtm/dtm.php?ID=ESP_036859_2020

Table S1: DTMs used in this work.

References

Chojnacki, M., M. E. Banks, L. K. Fenton, and A. C. Urso (2019), Boundary condition controls on the high-sand-flux regions of Mars, *Geology*, *47*(5), 1–4, doi:10.1130/G45793.1/4657361/g45793.pdf.

Runyon, K. D., N. T. Bridges, and C. E. Newman (2017), Martian sand sheet characterization and implications for formation: A case study, *Aeolian Res.*, *29*(April), 1–11, doi:10.1016/j.aeolia.2017.09.001.

Urso, A., M. Chojnacki, and D. A. Vaz (2018), Dune-Yardang Interactions in Becquerel Crater, Mars, *J. Geophys. Res. Planets*, 1–16, doi:10.1002/2017JE005465.
