# Supplementary figures and images for: Megaripple Migration on Mars
Source: J Geophys Res Planets. 2020 Jul 29;125(8):e2020JE006446. doi: 10.1029/2020JE006446 (PMC7583471; doi:10.1029/2020JE006446)

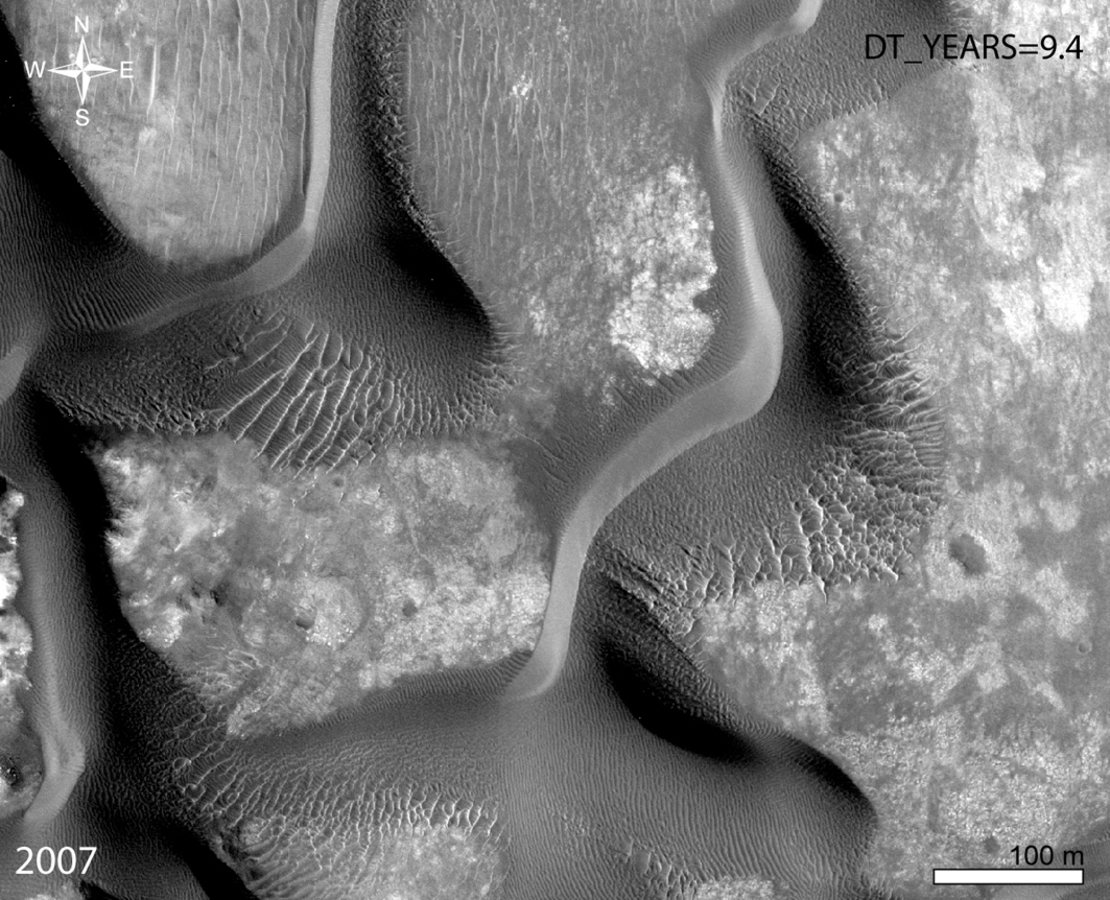

Supplement: Supplementary file 3 — Movie S1 [file JGRE-125-e2020JE006446-s003.gif]

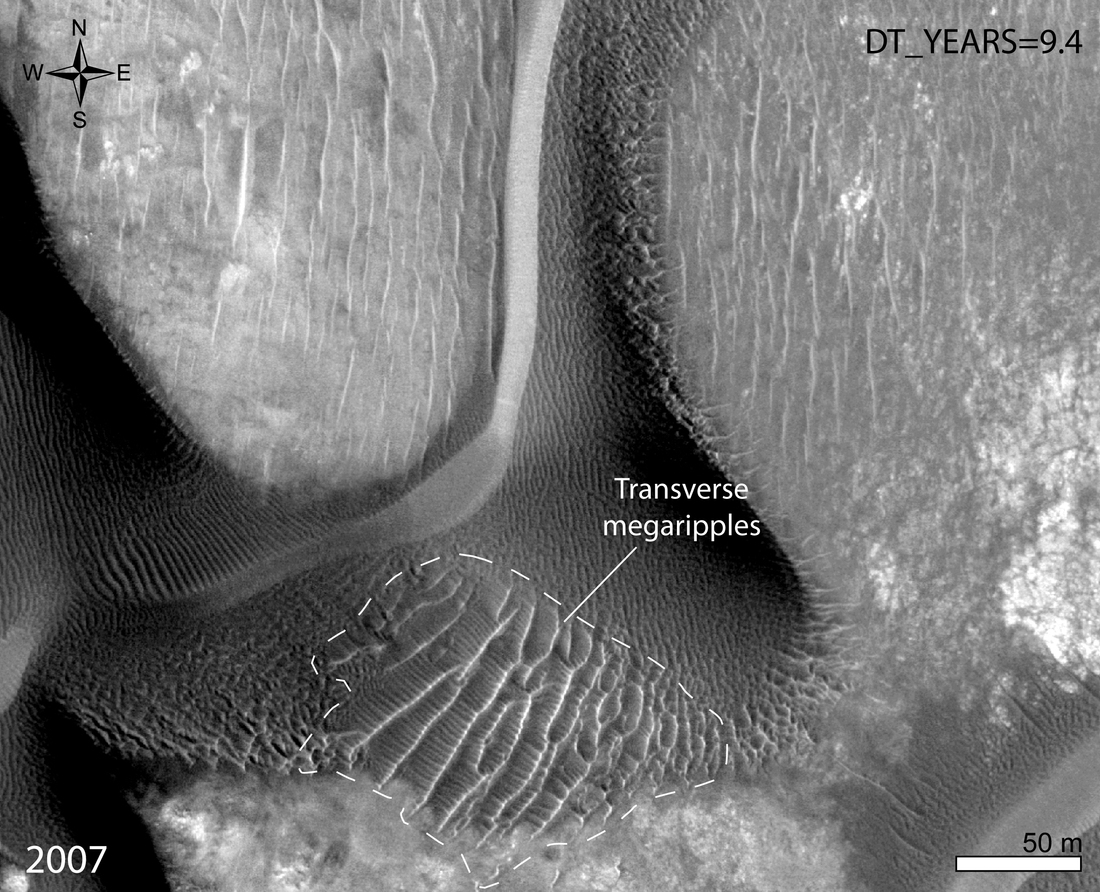

Supplement: Supplementary file 4 — Movie S2 [file JGRE-125-e2020JE006446-s004.gif]

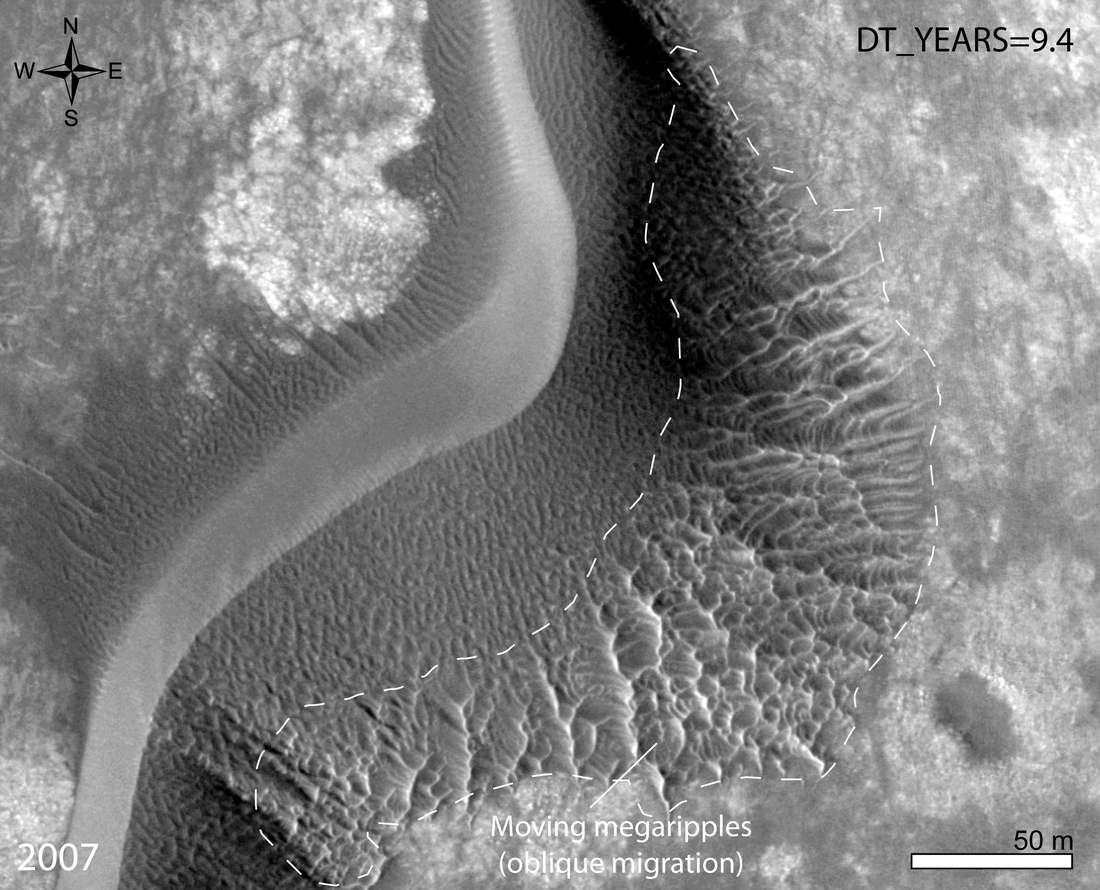

Supplement: Supplementary file 5 — Movie S3 [file JGRE-125-e2020JE006446-s005.gif]

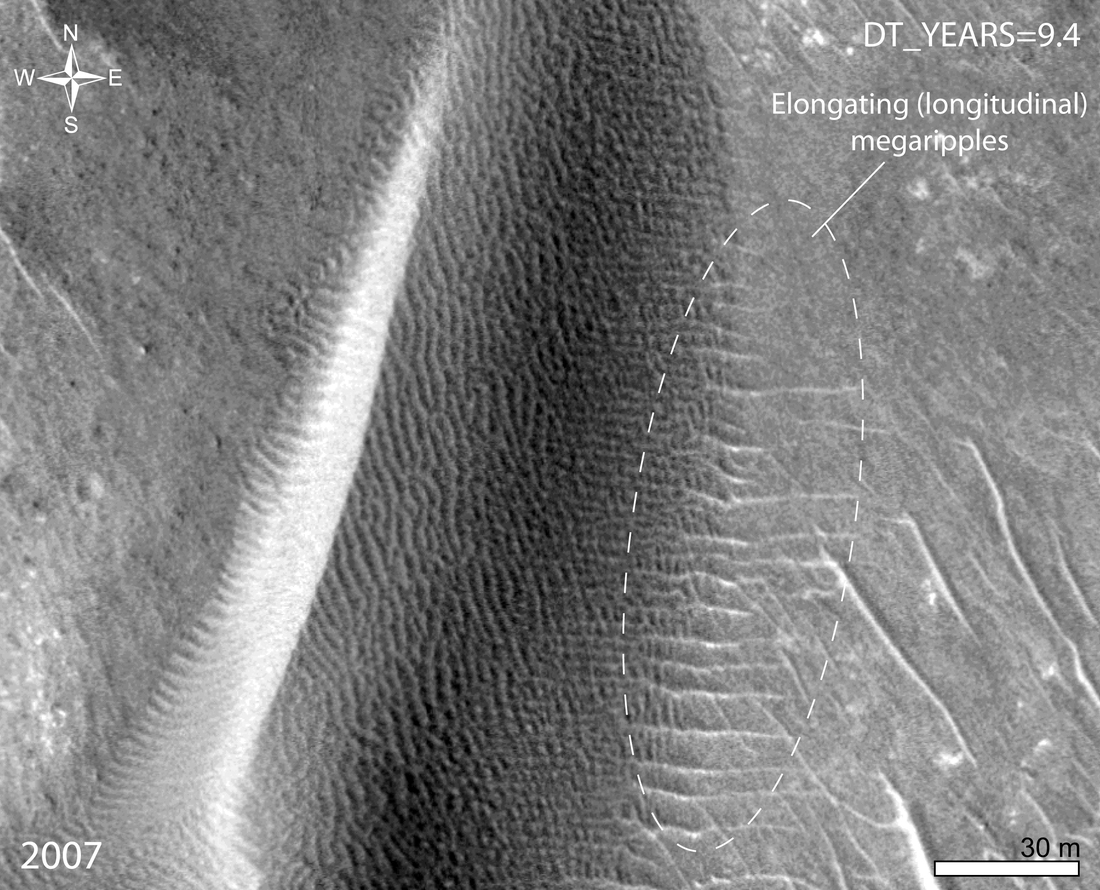

Supplement: Supplementary file 6 — Movie S4 [file JGRE-125-e2020JE006446-s006.gif]

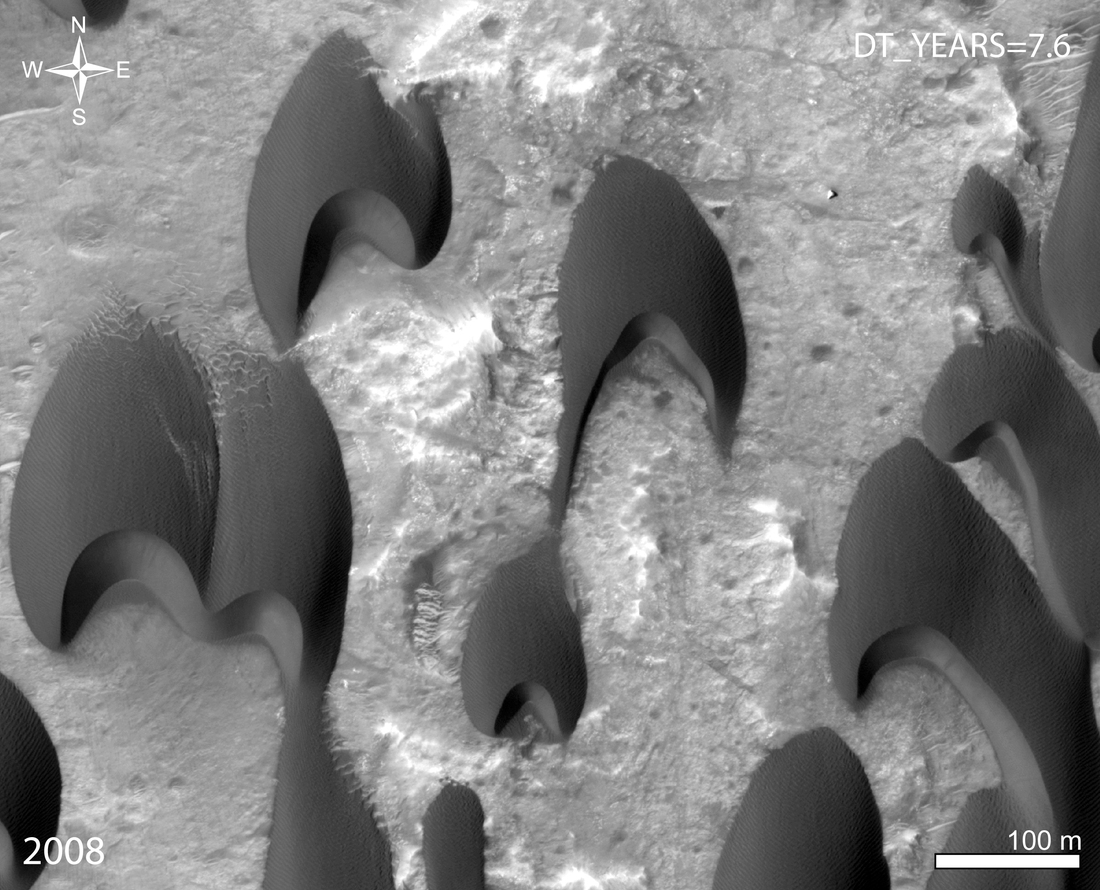

Supplement: Supplementary file 7 — Movie S5 [file JGRE-125-e2020JE006446-s007.gif]

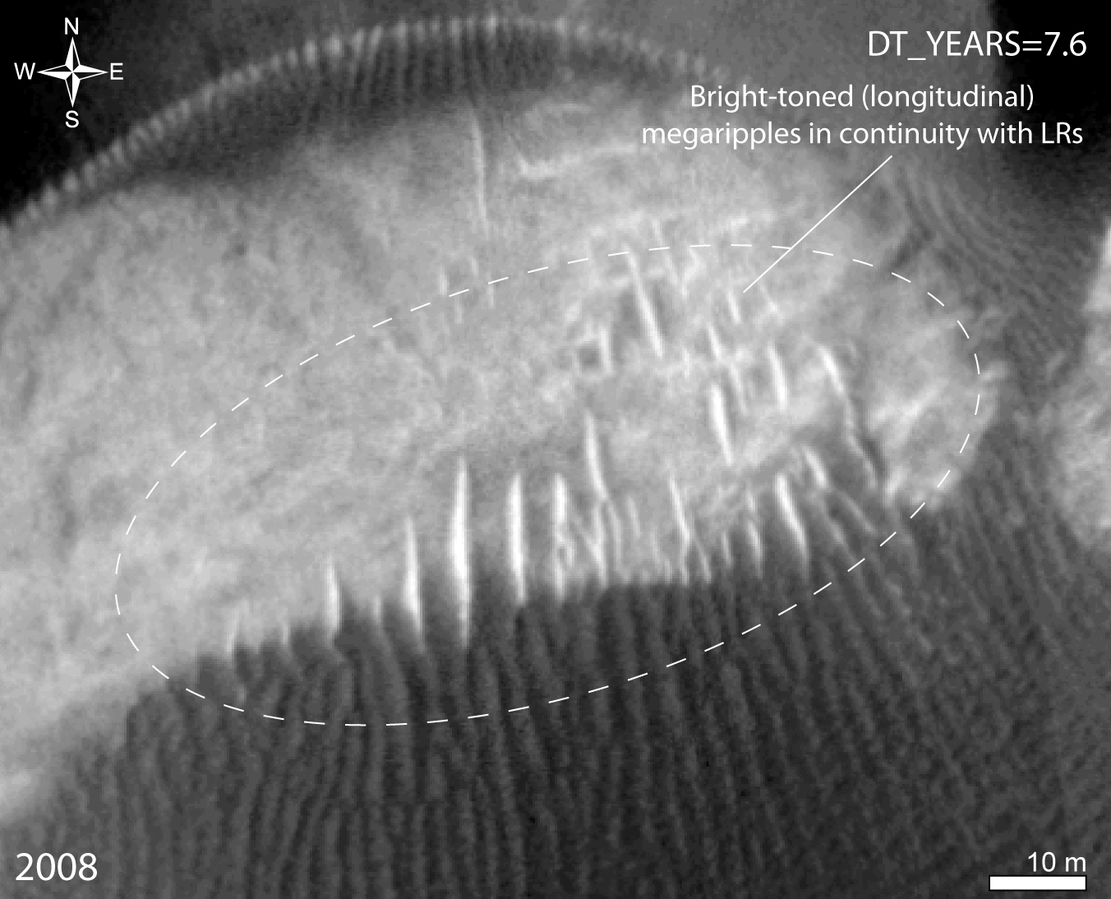

Supplement: Supplementary file 8 — Movie S6 [file JGRE-125-e2020JE006446-s008.gif]

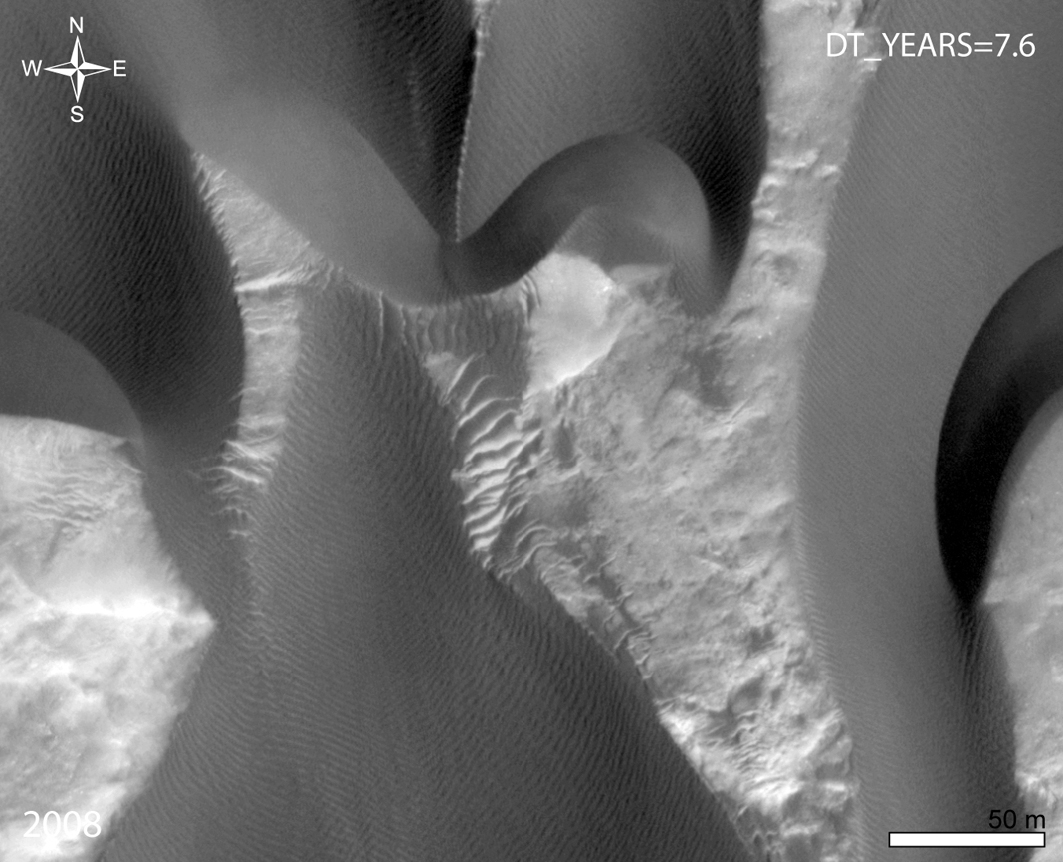

Supplement: Supplementary file 9 — Movie S7 [file JGRE-125-e2020JE006446-s009.gif]

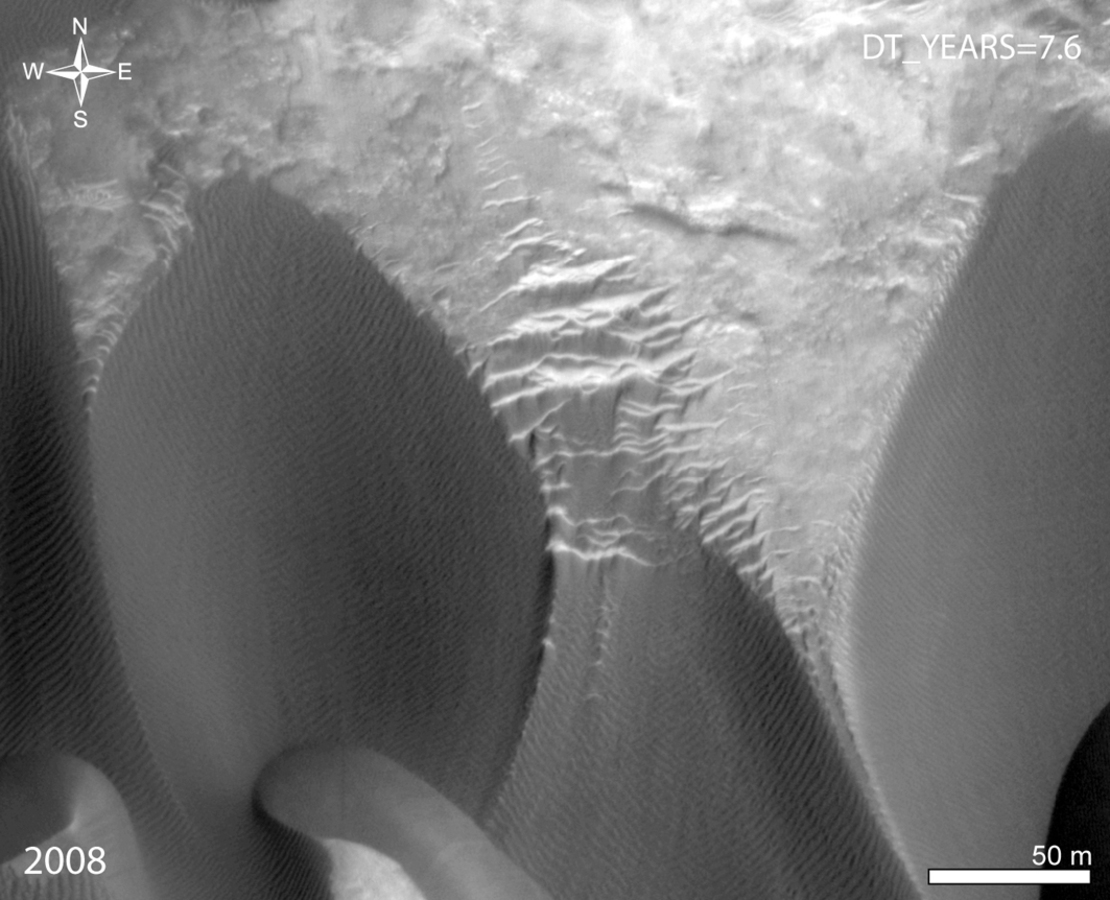

Supplement: Supplementary file 10 — Movie S8 [file JGRE-125-e2020JE006446-s010.gif]

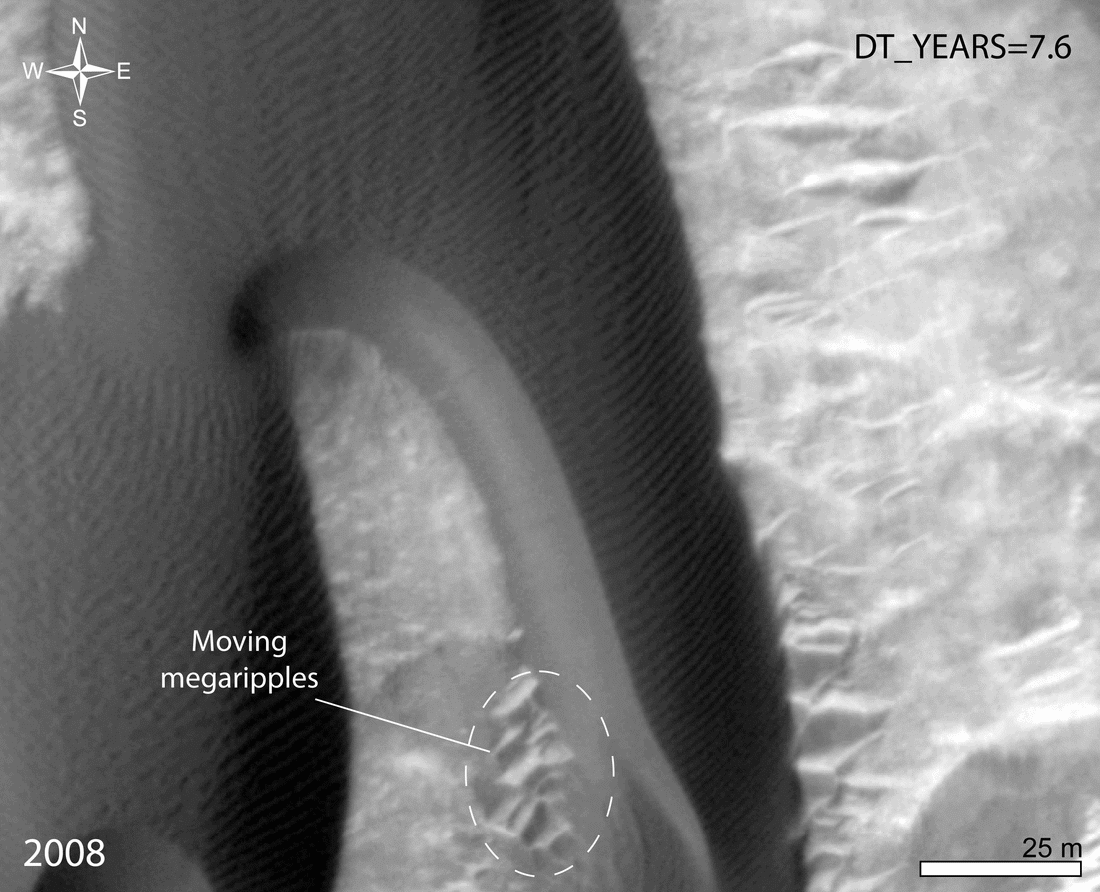

Supplement: Supplementary file 11 — Movie S9 [file JGRE-125-e2020JE006446-s011.gif]

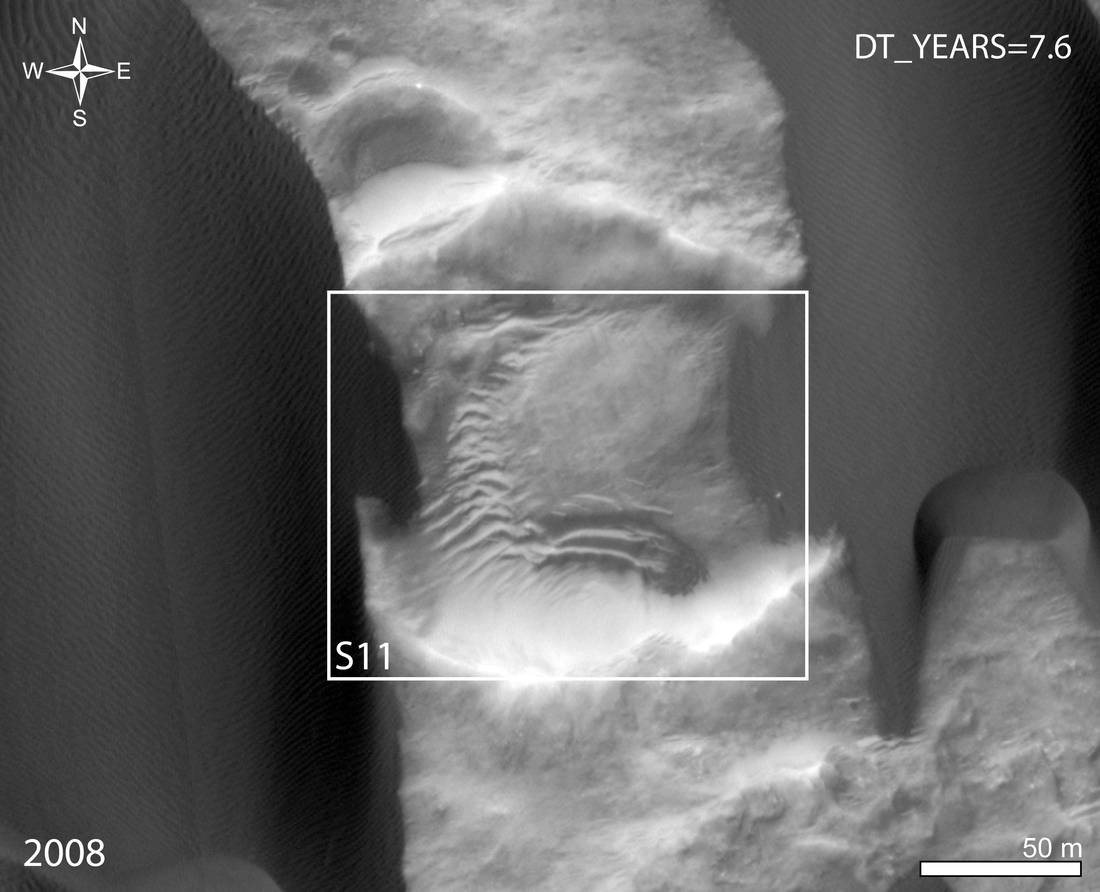

Supplement: Supplementary file 12 — Movie S10 [file JGRE-125-e2020JE006446-s012.gif]

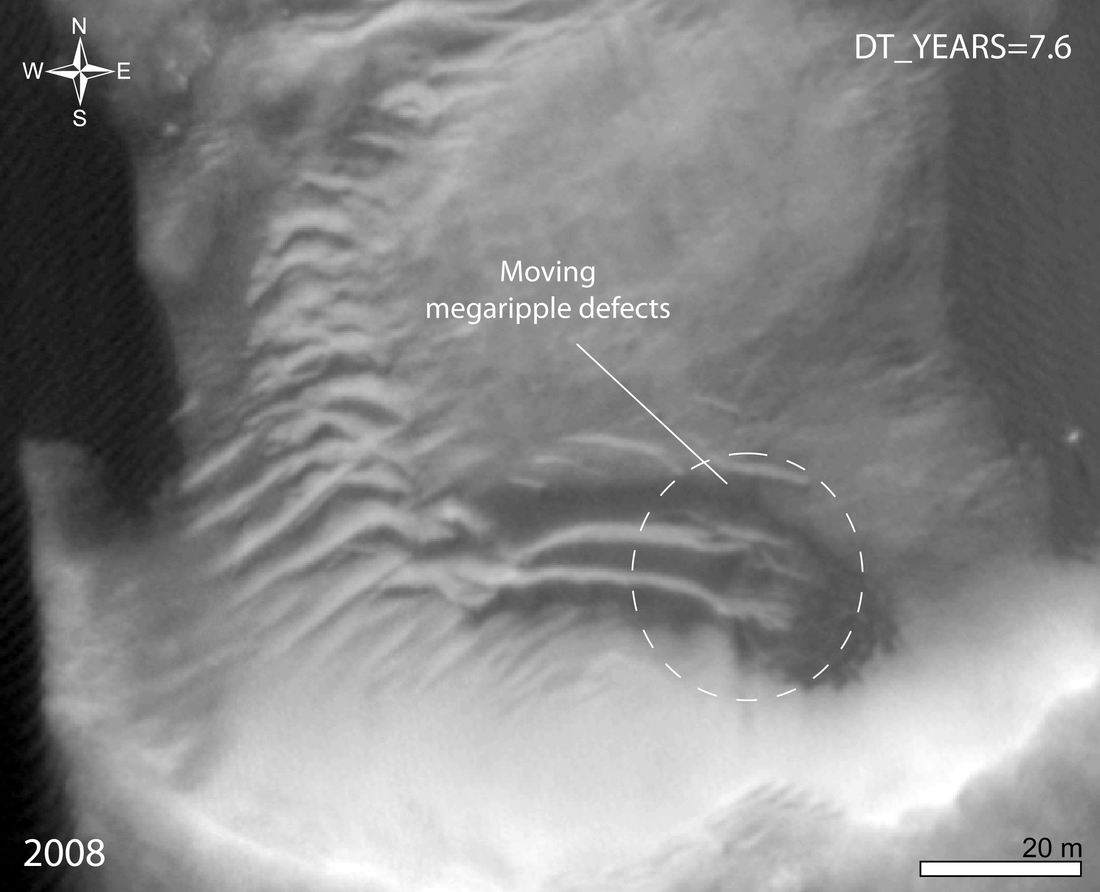

Supplement: Supplementary file 13 — Movie S11 [file JGRE-125-e2020JE006446-s013.gif]

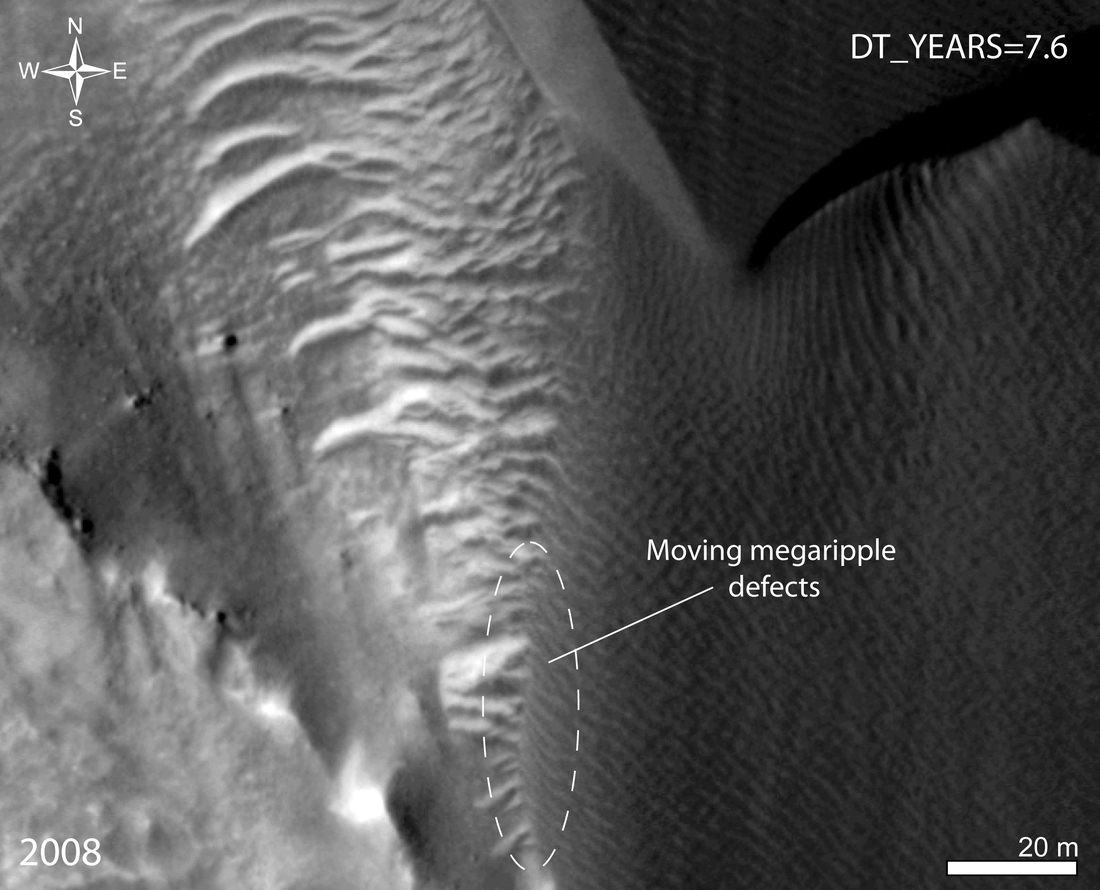

Supplement: Supplementary file 14 — Movie S12 [file JGRE-125-e2020JE006446-s014.gif]

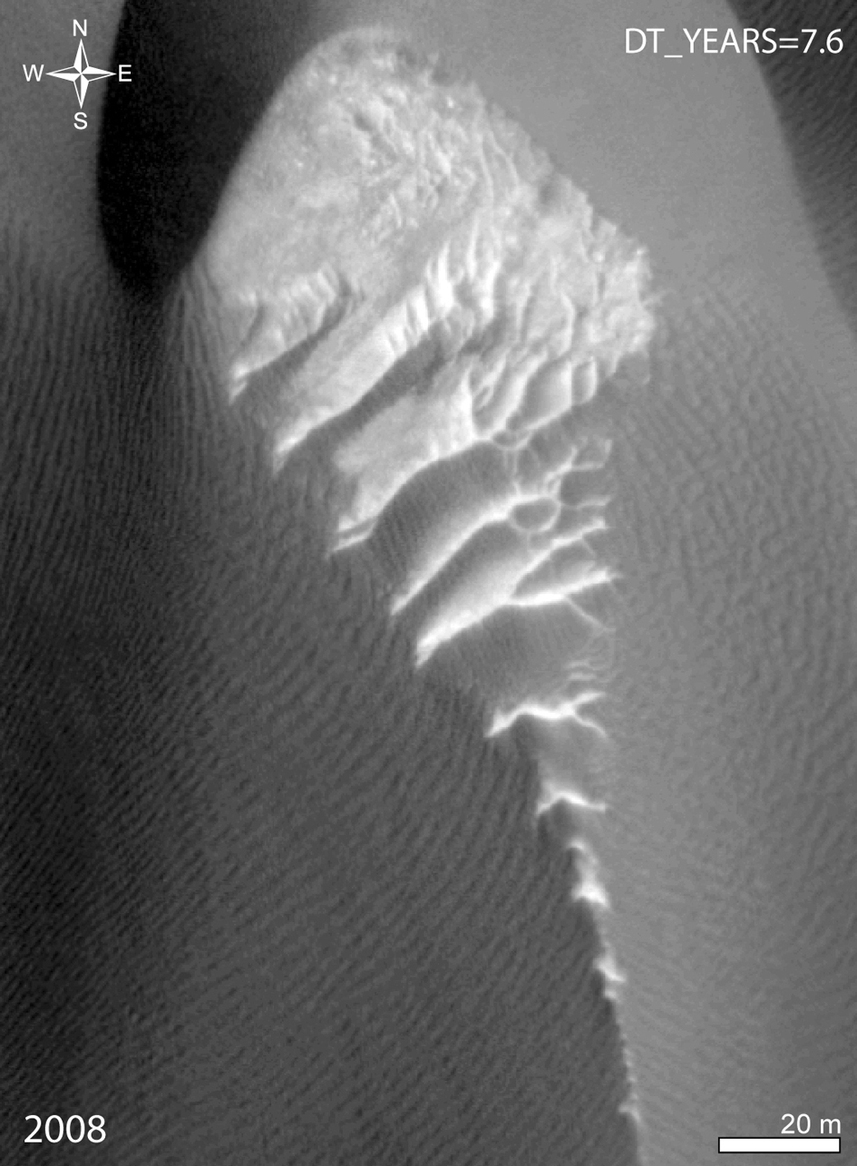

Supplement: Supplementary file 15 — Movie S13 [file JGRE-125-e2020JE006446-s015.gif]

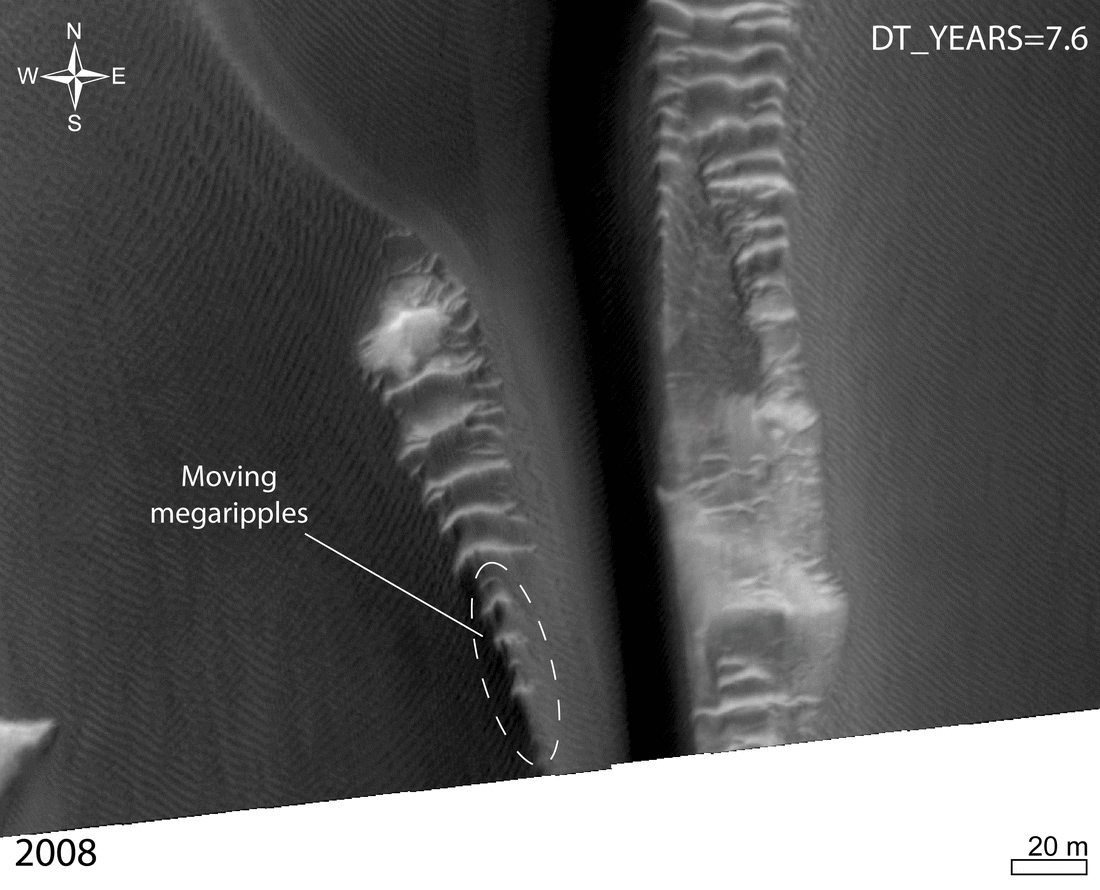

Supplement: Supplementary file 16 — Movie S14 [file JGRE-125-e2020JE006446-s016.gif]
